# Thermally regenerable montmorillonite/sand composite for the adsorptive removal of phenazopyridine from aqueous solutions

**DOI:** 10.1039/d6ra05599k

**Published:** 2026-08-05

**Authors:** Shatha Madi, Shaher Zyoud, Ameed Amireh, Samer H. Zyoud, Motasem Far, Dongjun Cha, Tae Woo Kim, Wasim A. Zyoud, Ahed H. Zyoud

**Affiliations:** a Master Program in Chemistry, Faculty of Graduate Studies, An-Najah National University Nablus Palestine; b Department of Building Engineering and Environment, Palestine Technical University (Kadoorie) Tulkarm Palestine; c Department of Chemistry, Faculty of Science, An-Najah National University Nablus Palestine; d Department of Mathematics and Sciences, Ajman University P.O. Box 346 Ajman United Arab Emirates; e Hydrogen Research Department, Korea Institute of Energy Research 152 Gajeong-ro, Yuseong-gu Daejeon 34129 Republic of Korea; f An-najah National University Faculty of Medicine and Health Sciences Palestine; g Center of Excellence in Materials Science and Nanotechnology (CEMSANT), An-Najah National University Nablus Palestine ahedzyoud@najah.edu +00970599353404

## Abstract

The presence of pharmaceuticals in aquatic ecosystems implies the need for efficient and sustainable treatment processes for their removal. This paper reports an innovative adsorption–regeneration process of a heat-resistant montmorillonite/sand composite (Mnt/sand) used to remove phenazopyridine (PHY) from aqueous media. Unlike other adsorption–thermolysis processes, which mostly use fine clay particles, this composite entraps the adsorptive Mnt phase within a fine sand matrix to improve the performance of the adsorbent. A maximum adsorption of 8.98 mg g^−1^ was achieved using the total weight of the Mnt/sand composite, whereas 44.90 mg g^−1^ was calculated on the basis of the weight of the active Mnt phase only. Kinetic studies revealed that the adsorption process occurs *via* pseudo-second-order kinetics (*R*^2^ = 0.997), suggesting that the rate-limiting step in the process is due to surface interactions. The equilibrium data followed the Langmuir isotherm model, indicating that monolayer adsorption occurred on homogeneous sites, whereas thermodynamic calculations revealed that the process was spontaneous and endothermic (Δ*G*° < 0, Δ*H*° > 0). Furthermore, the Mnt/sand composite was tested under continuous flow conditions using a fixed-bed column. In the case of the breakthrough run with a nominal *C*_0_ = 20 mg L^−1^, *Q* = 2 mL min^−1^ and *m* = 3.0 g, breakthrough was observed at 33.75 min, with a breakthrough capacity of 0.439 mg g^−1^. The kinetic parameters obtained from the Thomas model are *k*_Th_ = 5.14 × 10^−3^ L mg^−1^ min^−1^, *q*_Th_ = 0.758 mg g^−1^, and *R*^2^ = 0.963, representing an efficiency of the bed usage of 57.9% compared with the theoretical saturation capacity. The experiments on thermal regeneration revealed highly efficient restoration of the adsorption capacity of the material at approximately 550 °C, with stable performance throughout multiple cycles (87–90%). From the point of view of computational research, the adsorption of PHY molecules on Mnt is energetically favorable (*E*_ads_ = −0.1273 hartree = −334.25 kJ mol^−1^ = −3.46 eV), accompanied by structural modification of PHY, which could be helpful for the thermal degradation of PHY.

## Abstract 2

The presence of pharmaceuticals in aquatic ecosystems implies the need for efficient and sustainable treatment processes for their removal. Graphical abstract image partly generated by Microsoft Copilot.

## Graphical abstract

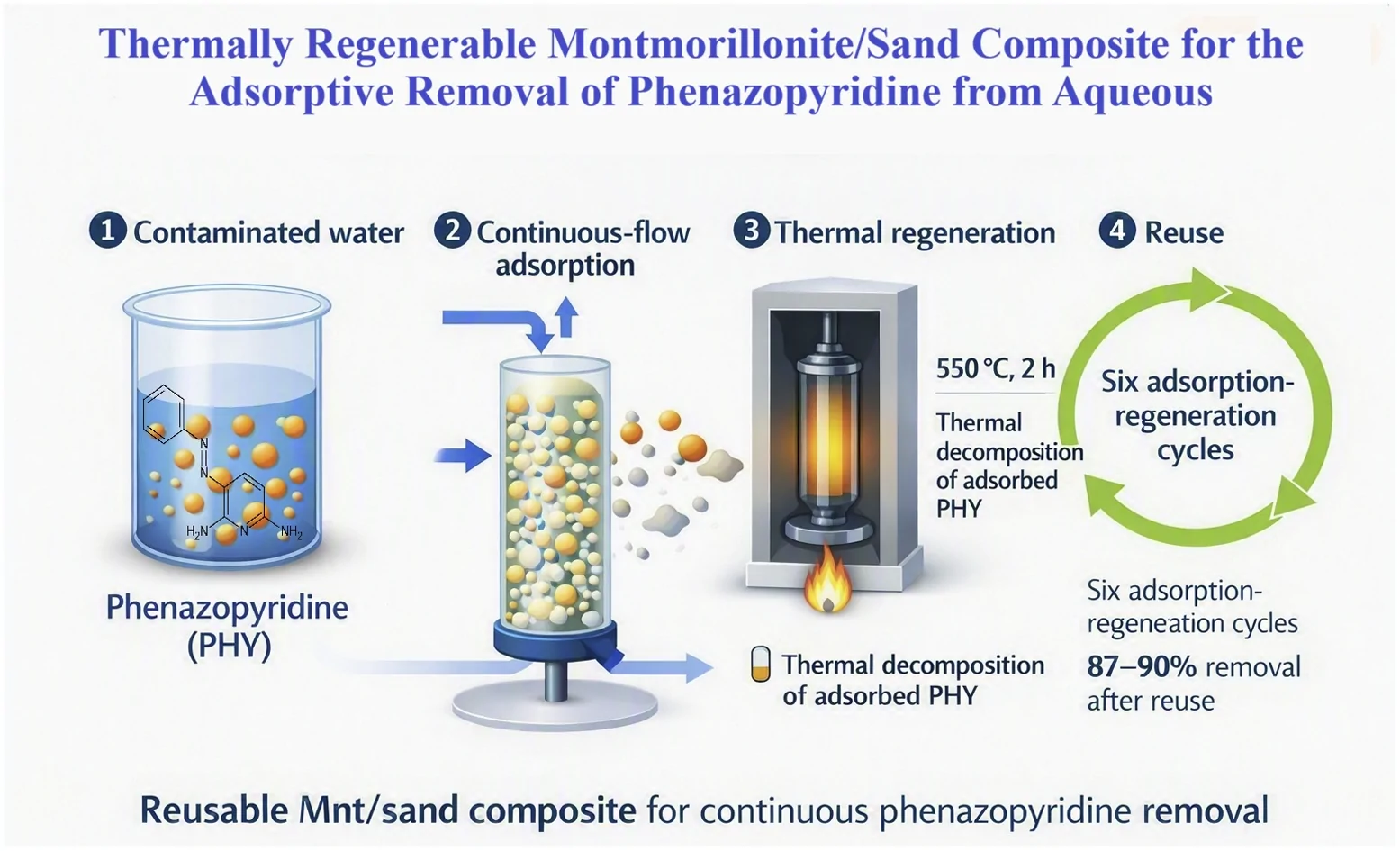

## 1. Introduction

Water contamination is one of the major environmental problems of the 21st century because of industrial development, population growth, and the use of numerous chemical compounds.^1^ Pharmaceutical compounds are among the categories of water pollutants that are gaining increasing interest because of their ongoing presence in aquatic environments, persistence and biological activity.^2,3^ In general, some pharmaceuticals do not undergo complete metabolism and can be excreted from organisms in their primary form and/or as metabolites *via* urine and feces.^4^ Pharmaceuticals can enter sewer systems and then wastewater treatment plants that are not prepared for removal from water.^5,6^ Therefore, pharmaceuticals can be discharged in natural water bodies as emerging pollutants.^7^

The problem of pharmaceuticals being detected in water environments is especially relevant because of the possible effects these contaminants have on aquatic organisms in terms of causing physiological and behavioral alterations, hormonal imbalances, and reproductive disorders.^8,9^ Additionally, some chemicals can accumulate in sediments and organisms and thus pose a long-term threat to the environment. Furthermore, the continuous discharge of substances with biological activity can lead to the development of antimicrobial resistance, which poses a serious threat to public health globally.^10^ Moreover, the chemical stability and low biodegradability of these compounds make the treatment process difficult.^11,12^

Wastewater treatment typically requires several physical, chemical, and biological processes on the basis of the types and levels of contaminants present and the desired outcome. Some of these techniques include chemical precipitation, coagulation–flocculation, flotation, oxidation–reduction, ion exchange, solvent extraction, electrodeposition, membrane separation, reverse osmosis, biosorption, and adsorption.^13–16^ Membrane-based processes are also strongly influenced by membrane charge, electrolyte composition, ion valence, and membrane potential in charged porous systems.^17^ All of these methods have strengths and weaknesses. For instance, the membrane separation process may offer high separation efficiency, but it may still be prone to fouling, high-pressure operation, and high costs. Similarly, chemical precipitation and oxidation–reduction techniques may effectively treat particular contaminants but still require SI chemicals and generate secondary sludge. Among these processes, adsorption is widely used because of its simplicity, easy control, relatively low cost, and ability to remove trace organics from aqueous streams.^18,19^

Phenazopyridine (PHY) is a urinary analgesic that is widely employed in the management of pain, burning, irritation, and other symptoms of UTIs and other UT disorders.^20–22^ Considering its medical usage and partial metabolism, some of the consumed PHY is discharged in wastewater as either nonmetabolized PHY or as a metabolite. Chemically, PHY comprises aromatic rings and nitrogen-bearing groups, contributing to high chemical stability and the persistence of the compound.^23,24^ In addition, its deep coloring has a negative effect on the quality and appearance of water. Thus, PHY can be regarded as a model substance for studying water purification from persistent pharmaceutical pollutants.

Materials employed as adsorbents for water treatment include various natural, synthetic, and fabricated solid compounds, such as activated carbon, carbonaceous materials, clay minerals, zeolites, metal–organic frameworks, metal oxides, agricultural waste products, biosorbents, polymeric adsorbents, and composite materials.^25–31^ Such materials can eliminate pollutants through different methods, such as electrostatic interactions, ion exchange, hydrogen bond formation, surface complexation, π-interactions, pore filling, and van der Waals forces. The selection of a suitable adsorbent depends not only on the adsorption capacity and selectivity but also on the cost, availability, mechanical stability, regeneration ability, and applicability in batch or continuous-flow systems.

Clay-based adsorbents such as montmorillonite (Mnt), whose layered structure, high surface area, and high cation-exchange capacity make strong interactions with organic contaminants possible,^25,26,32–35^ have been widely studied. The practical application of Mnt in fine form is hampered by its tendency to remain suspended in aqueous media because of the formation of stable suspensions, which prevents the separation of the solid and liquid phases and causes difficulties in adsorbent recovery. Furthermore, the presence of fine clay particles in fixed-bed columns can lead to poor permeability and blockage of the bed.

To eliminate these disadvantages, sand was used as a supporting matrix to produce a montmorillonite/sand (Mnt/sand) composite. As an inert material with a low adsorption capacity, sand has good mechanical strength, chemical stability, and permeability. Its inclusion helps create granular composites with better handling, recoverability, mechanical properties, and flow behavior in fixed beds. Hence, in the created composite, Mnt acts as an adsorptive component, whereas sand acts as an inert support. In this way, the Mnt/sand composite represents a combination of the adsorption ability of Mnt and the operational characteristics required for practical treatment.^16,36^

Although adsorption is effective, the regeneration of saturated adsorbents remains a major concern because chemical desorption may generate secondary effluents and increase treatment costs. Thermal regeneration offers an alternative by decomposing adsorbed organic contaminants and restoring adsorption sites without chemical regenerants. Earlier studies by Zyoud and coworkers developed the concept of adsorption–thermolysis using thermally stable montmorillonite, modified natural clay, and kaolinite-based adsorbents for tetracycline, methyl orange, phenazopyridine, other organic pollutants, and nitrate-containing systems.^32,33,35,37,38^ However, these studies relied mainly on fine clay particles in batch systems, where difficult recovery, particle loss, poor permeability, and bed clogging limit continuous-flow application. The present work therefore advances beyond merely using PHY as another model contaminant by immobilizing active Mnt on a coarser sand support to produce a thermally regenerable and column-compatible composite. This design improves handling, recovery, permeability, and fixed-bed operation while retaining the adsorption contribution of Mnt, thereby extending adsorption–thermolysis from fine-clay batch systems toward continuous-flow water treatment.

Therefore, this study aims to prepare a thermally stable Mnt/sand composite and evaluate its performance for PHY removal from aqueous solutions using an integrated adsorption–thermal regeneration approach. Unlike earlier fine-clay batch systems, the present system was evaluated under both batch and continuous-flow conditions. Adsorption behavior was investigated through equilibrium, kinetic, thermodynamic, diffusion, regeneration, and fixed-bed breakthrough analyses. Furthermore, density functional theory (DFT) was applied to explain the PHY interaction on the Mnt surface. Overall, this study provides insight into the practical use of a regenerable clay/sand system for pharmaceutical-contaminated water treatment and the development of adsorption–thermolysis technology for continuous-flow processes.

## 2. Materials and methods

### 2.1 Chemicals and reagents

All the chemicals used in this study were of analytical grade and were used without further purification. PHY (CAS: 136-40-3), Mnt clay (CAS: 1318-93-0), and potassium chloride (KCl) (CAS: 7447-40-7) were purchased from Sigma-Aldrich. Sodium hydroxide (NaOH), hydrochloric acid (HCl), sulfuric acid (H_2_SO_4_), and nitric acid (HNO_3_) were obtained from CS Chemical Company. Distilled water was used as the solvent for the preparation of all aqueous solutions throughout the experiments. In this study, PHY was selected as the model pharmaceutical contaminant (adsorbate), while a Mnt/sand composite was prepared and employed as the adsorbent material. The sand used as the supporting matrix for Mnt was collected from the coastal shore of Ashdod in Palestine.

### 2.2 Instrumentation

The concentration of PHY in water was measured with a UV-vis spectrometer (Shimadzu UV-1800). The pH of the solutions was measured by a pH meter (Jenway Model 3510). Adsorption experiments were performed in batch mode with a water bath shaker from Lab Tech (LSB-015S) to keep the temperature and agitation frequency constant during the adsorption process. Following adsorption, the adsorbent was separated from the solution by means of centrifugation with a LABTRON LLS-A12 centrifuge. Thermal regeneration of the contaminated adsorbent materials was performed by placing the spent adsorbent in a bench-top muffle furnace (MRC) to thermally breakdown the adsorbed organic pollutants. The physicochemical characteristics of the adsorbent samples were investigated by different analytical methods. To identify the crystalline structure and phase composition of the samples, X-ray diffraction (XRD) scans were carried out with a PANalytical XPert PRO diffractometer fitted with CuK radiation (1. 5418). The surface morphology and microstructural details were inspected with scanning electron microscopy (SEM) (JEOL-EO). Fourier transform infrared (FT-IR) spectroscopy was performed on a Thermo Scientific Nicolet iS5 spectrometer at An-Najah National University to determine the functional groups on the surface of the adsorbent and to explore the interactions of PHY with the adsorbent. In addition, thermogravimetric analysis (TGA) using a SANAF TGA analyzer was performed to assess the thermal stability of the synthesized adsorbent and determine the thermal decomposition temperature of PHY, which is a key factor for selecting suitable conditions for the thermal regeneration process.

### 2.3 Preparation of Mnt/sand composite

Ashdod beach sand was sampled and sieved using standard U.S. meshes to obtain fractions of 600–1180 µm. Large impurities and stones were separated using mesh No. 16 (>2.36 mm). Afterward, the sand was further sieved through mesh No. 30 to separate the required particle-size fraction. Approximately 300 g of sieved sand was thoroughly removed using distilled water to eliminate any surface contamination. To prepare highly pure silica sand, the product was chemically treated using an acid washing process. Dilute nitric acid (1 : 2) was slowly poured on the sand, followed by the addition of equal amounts of dilute hydrochloric acid to eliminate metal oxides, carbonates, and organic impurities. The product was heated for 2 hours with constant stirring and then allowed to rest overnight at room temperature until the reaction was complete. After the acid treatment, the sand was repeatedly washed with distilled water to a neutral pH and then dried in an oven at 100 °C for 2 hours. Composite absorbents were prepared by mixing Mnt and pure silica sand in 1 : 4 weight ratios.^39,40^ In brief, 20 g of Mnt was stirred into 80 g of synthesized silica sand in a 250 mL beaker. Afterward, distilled water was carefully added to form a homogeneous granular paste. The Mnt/sand composite was subsequently calcined at 550 °C for 2 h to obtain a thermally stable Mnt/sand composite for the column-type water purification process.^41,42^

To estimate the reproducibility of the preparation, Mnt/sand composite preparation was carried out in three batches under identical experimental conditions. In each batch, 20.0 g of Mnt was mixed with 80.0 g of purified sand, which corresponded to a total dry solid mass of 100.0 g. After calcination at 550 °C for 2 h, the dry composite masses were 95.0, 93.0, and 94.0 g for the first, second, and third batches, respectively. The preparation yield was determined as follows: preparation yield (%) = [final dry composite mass/initial dry solid mass] × 100. Thus, the preparation yields were 95.0%, 93.0%, and 94.0%, with an average yield of 94.0 ± 1.0% (*n* = 3) and an RSD of 1.06%. The small amount of weight loss that occurred during the process of preparation was due to slight attachment of the wet composite paste to the preparation/calcination vessel. The relatively low RSD value confirms the high reproducibility of the laboratory-scale preparation process of Mnt/sand.

To conduct batch adsorption tests, the same method of preparation mentioned above was used. Notably, in the case of the batch adsorption test, no need existed to control the particle size accurately, so the sieving step was skipped. In addition, smaller particles were chosen to ensure better contact between the adsorbent and the solution.

### 2.4 Determination of the point of zero charge (pH_pzc_)

The point of zero charge (pH_pzc_) of the Mnt/sand composite was determined using the pH drift technique. The solution used in this experiment as the background electrolyte was 0.1 M KCl. This solution was prepared by mixing 3.72 g of KCl dissolved in 500 mL of distilled water. Solutions with various initial pH values (2, 3, 4, 6, 8, 10, and 12) were prepared by adjusting the pH of the solution with HCl or NaOH. Afterward, 50 mL of each of the solutions was placed in individual glass bottles, where 0.10 g of Mnt/sand composite was added to each. After the mixture was shaken at 200 rpm and maintained at 25 °C for 24 h, the final pH values were measured, and the point of zero charge was calculated by plotting the ΔpH (the change in pH, pH_final_ − pH_initial_) against the initial pH value.^27,43^

### 2.5 Characterization of the adsorbent materials

The prepared adsorbent materials were characterized using several analytical techniques to evaluate their structure, morphology, functional groups, thermal stability, and surface area. Fourier transform infrared spectroscopy (FT-IR) was used to identify the functional groups of Mnt/sand, pure PHY, and the PHY-loaded Mnt/sand composite after adsorption. Regenerated adsorbents were also analyzed by FT-IR to verify the removal of adsorbed organic residues after thermal regeneration. X-ray diffraction (XRD) was performed to assess the crystalline structure and mineral composition of Mnt and the Mnt/sand composite. Scanning electron microscopy (SEM) was used to examine the surface morphology and microstructure of the adsorbent materials before and after adsorption. Thermogravimetric analysis (TGA) was carried out to evaluate the thermal stability of the Mnt/sand composite and to determine the thermal decomposition behavior of PHY, which is important for selecting suitable thermal regeneration conditions.^33^ Specific surface area measurements were also performed for sand, Mnt, and the Mnt/sand composite using the Brunauer–Emmett–Teller (BET) method. The measurements were conducted using a manually prepared vacuum-line system. Prior to analysis, the samples were dried to remove physically adsorbed moisture, and the specific surface area values were calculated according to the BET approach.

### 2.6 Adsorption experiments

All the quantitative adsorption, regeneration, and continuous-flow experiments were carried out in triplicate under identical conditions. The reported values represent the average of the triplicate measurements, and the error bars shown at each point in the figure profiles correspond to the standard deviation of the replicate measurements.

#### 2.6.1 Batch adsorption experiments

Batch adsorption experiments were conducted to evaluate the adsorption performance of the Mnt/sand composite for PHY removal from aqueous solutions. In each experiment, 100 mL of PHY solution was placed in a capped vessel and placed in contact with the required mass of adsorbent. The vessels were sealed and shaken in a water bath shaker at 200 rpm for 1 h to ensure sufficient contact between the adsorbent and the contaminant. Samples were withdrawn at 10 min intervals during kinetic experiments. After adsorption, the adsorbent was separated from the solution by centrifugation, and the remaining PHY concentration in the supernatant was determined by UV-vis spectrophotometry at a maximum absorbance wavelength (*λ*_max_) of 430 nm. The effects of the initial PHY concentration (10–50 mg L^−1^), solution pH (3.5–10), temperature (10–50 °C), and adsorbent dosage (0.2–1.0 g) were investigated. Adsorption capacity and removal efficiency were calculated from the difference between the initial and equilibrium PHY concentrations. After adsorption, the Mnt/sand composite was heated in a muffle furnace at 550 °C for 2 h to decompose the adsorbed organic residues and restore the material for reuse.^33,42^

#### 2.6.2 Continuous column adsorption experiments

Continuous adsorption experiments were performed using a fixed-bed glass column with an outer diameter of 12 mm, an inner diameter of 10 mm, and a length of 10 cm. The column was packed with the prepared montmorillonite/sand (Mnt/sand) composite. To vary the bed heights in this experiment, two different bed heights (4 and 8 cm) were attained by employing different diameters for the columns while keeping the amount of adsorbent constant, that is, 3 g. To keep the bed packing consistent, glass wool pads were added at each end of the column.

The influent solution, which contained PHY, was injected into the column *via* a peristaltic pump (Masterflex L/S, Cole-Parmer, USA) at flow rates between 2 and 10 mL min^−1^. The samples taken regularly at 10–20 min intervals were analyzed using a UV-vis spectrophotometer at a *λ*_max_ of 430 nm to measure the remaining PHY concentration.

The effects of the initial concentration of PHY (20–50 mg L^−1^), flow rate (2–10 mL min^−1^), bed height (4–8 cm) and temperature (10–50 °C) were studied. A breakthrough curve was constructed by plotting *C*_*t*_/*C*_0_*vs.* time, where *C*_0_ is the initial concentration and *C*_*t*_ is the time-dependent effluent concentration. The value of the breakthrough time (*t*_b_) was determined when *C*_*t*_/*C*_0_ = 0.10, whereas exhaustion was defined when *C*_*t*_/*C*_0_ = 0.95.

The parameters for column operation were estimated from the numerical breakthrough curve. The treated volume at breakthrough, adsorption capacity at breakthrough, total column capacity, and efficiency of column utilization were estimated according to the definitions and integration process explained in SI Section S5. Since the recorded breakthrough curve does not reach *C*_*t*_/*C*_0_ = 0.95, no exhaustion time was found in the experiments. Instead, the capacity estimated from the Thomas model is provided as the saturation/exhaustion capacity obtained from the model.

The Thomas model was chosen as the typical fixed-bed model for breakthrough behavior. Details of the equations, numerical values, regression graph, and calculation process can be found in SI Section S5. Only major findings for fixed-bed operations are reported and discussed in this manuscript.

For each continuous-flow experiment, a certain volume of influent PHY solution was completely passed through the packed bed under the set operating conditions. The residual PHY concentration was measured using a UV-visible Spectrophotometer after the entire volume of the influent solution was passed through the column. The overall removal efficiency (%) was estimated using the difference between the initial influent concentration (*C*_0_) and the effluent concentration (*C*_*t*_).

### 2.7 Molecular simulation study

DFT calculations were carried out to analyze the interaction behavior between the PHY molecules and the montmorillonite surface. First, structural optimization and conformer sampling were performed using the semiempirical GFN*n*-xTB approach to find energetically optimal adsorption modes. After that, further refinement of the DFT calculations was performed using the DMol^3^ module of the Materials Studio software package (BIOVIA, Dassault Systèmes, USA).

The DFT calculations were carried out within the periodic boundary conditions by applying the GGA and Perdew–Burke–Ernzerhof (PBE) exchange–correlation functional. The calculations were carried out by the double numerical basis set with polarization functions (DNP). Energy minimization was performed until the criteria of energy convergence, force convergence, and displacement convergence were met.

In the current study, DFT calculations were performed without dispersion correction. Therefore, the computational results should be treated mainly as a mechanistic explanation of the adsorption geometry, intermolecular interactions, perturbations of bond lengths due to the adsorption, and favorable adsorption energy based on the selected model. To obtain quantitative data concerning the influence of long-range van der Waals dispersion interactions on the Mnt surface, dispersion-corrected calculations using PBE-D, PBE-D3 or similar approaches should be carried out in the future.

The optimized adsorption structures were examined to determine possible adsorption geometries, intermolecular interactions, changes in PHY bond lengths due to the adsorption effect, and the adsorption energy of PHY on the Mnt surface. The adsorption energy was determined using the following formula: *E*_ads_ = *E*_Mnt/PHY_ − (*E*_Mnt_ + *E*_PHY_), where *E*_Mnt/PHY_, *E*_Mnt_, and *E*_PHY_ are the total energies of the optimized structure of the PHY/Mnt adsorption complex, the clean Mnt surface, and the free PHY molecules, respectively. A negative value of *E*_ads_ indicates favorable adsorption.

## 3. Results and discussion

### 3.1 Characterization of the adsorbent and adsorbent

The physical structure and morphology of the developed Mnt/sand composites were characterized by means of a few analytical methods, such as X-ray diffraction (XRD), scanning electron microscopy (SEM), and thermogravimetric analysis (TGA). XRD was performed to determine the crystalline phases contained in the sand, Mnt, and developed composite samples.

**Fig. 1:**
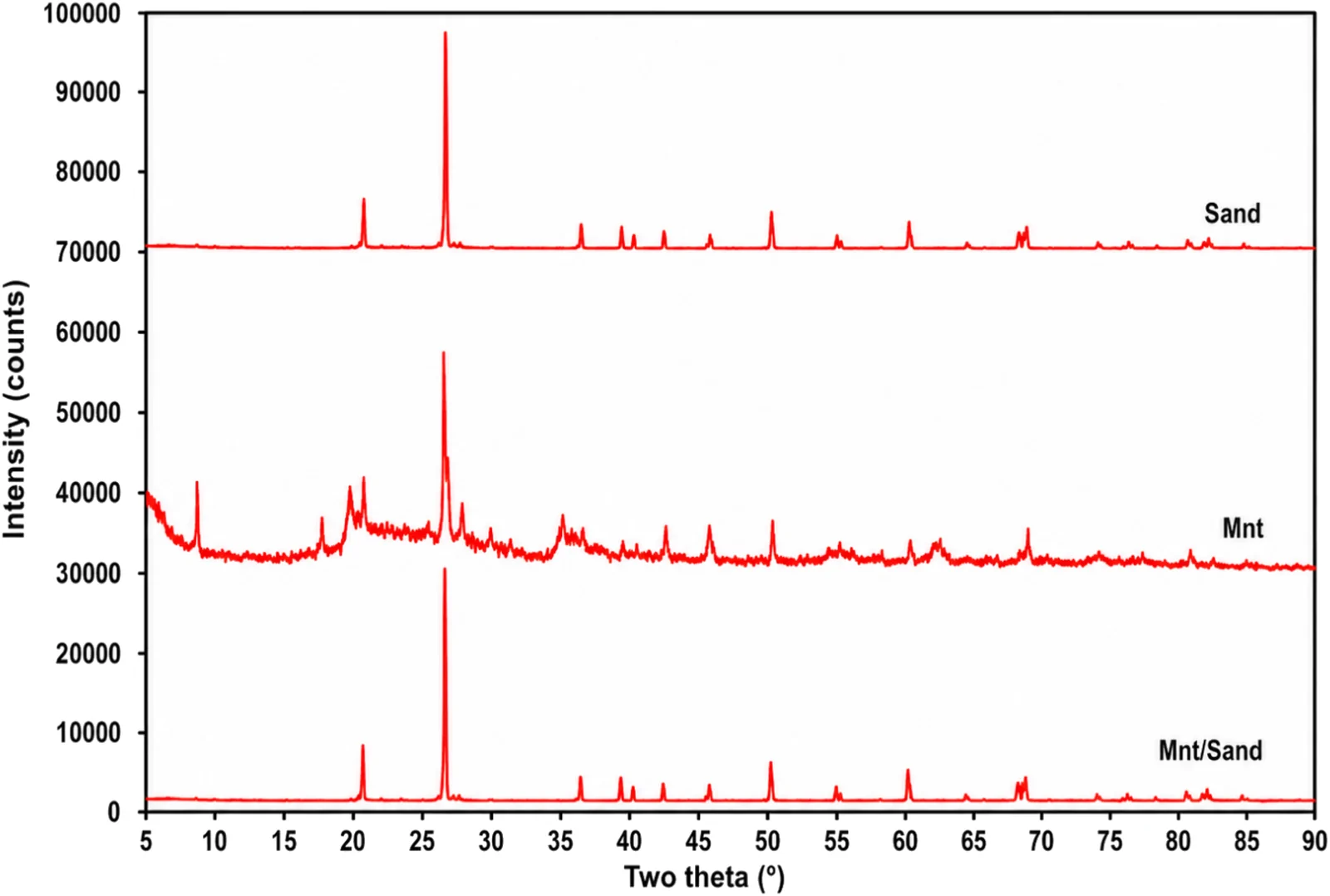
Combined XRD patterns of the sand, Mnt, and Mnt/sand composite. The patterns are vertically shifted for clarity.

The XRD patterns of sand, Mnt, and Mnt/sand are shown in Fig. 1. As shown in the figure, the sand sample has strong peaks at 2*θ* angles of 20.68°, 26.48°, 36.36°, 39.32°, 50.02°, and 68.18°, which correspond to the crystalline phases of silica. The typical reflections of Mnt were 8.74°, 20.68°, 26.48°, 34.92°, 49.92°, and 68.16°. When the composite adsorbent was prepared, the resulting diffraction pattern indicated signatures for both materials, thus confirming the successful synthesis of the composite material.^44^ The most significant peak at approximately 26° (2*θ*), which is characteristic of quartz, was observed for both sand and Mnt and was stronger for the composite material. These findings suggest that the formation of the composite material occurred mainly through physical mixing of the two materials rather than through any chemical reaction. In this case, the sand simply acts as a mechanical support for Mnt, which is the adsorbing phase.

**Fig. 2:**
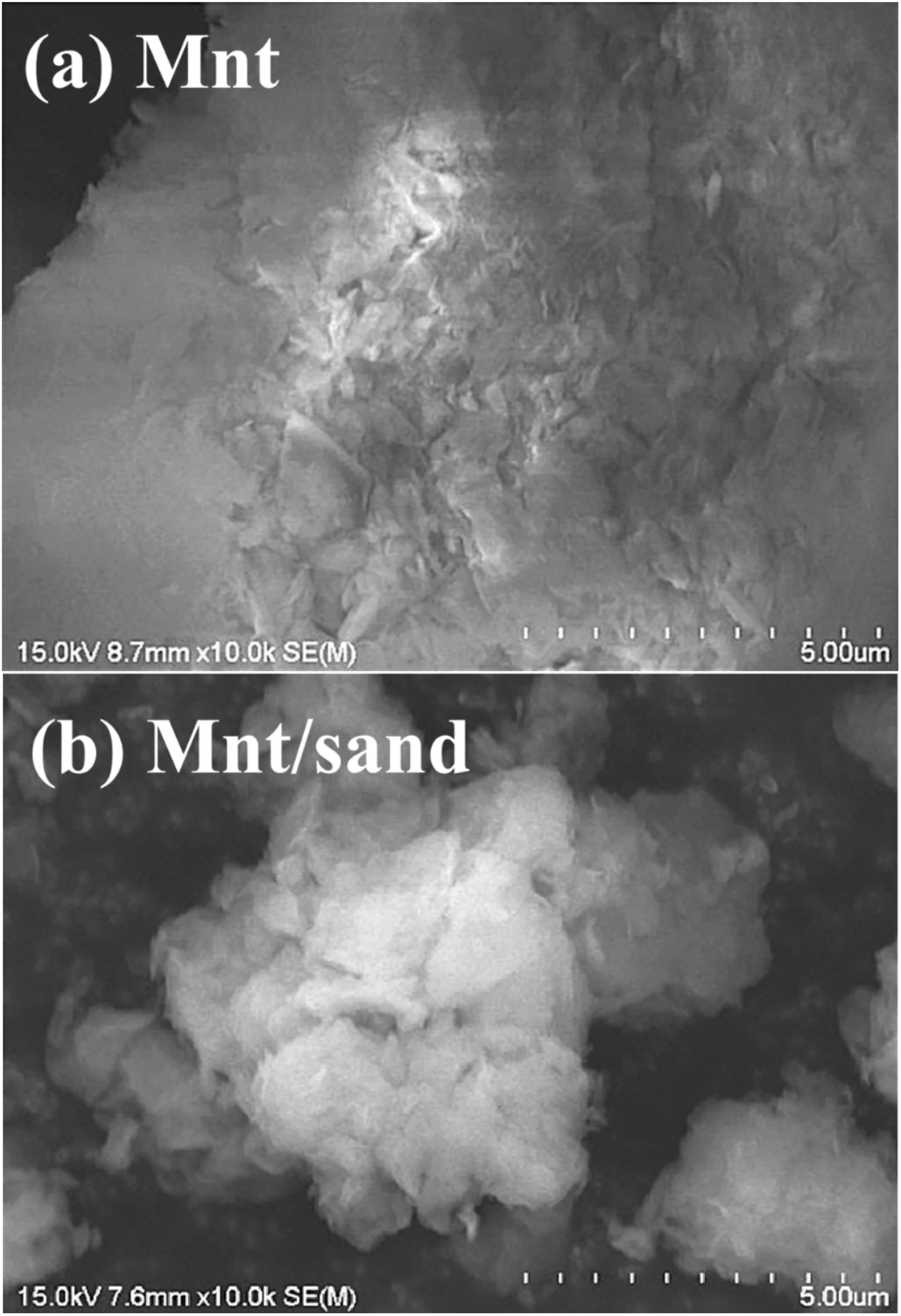
SEM images of (a) Mnt and (b) the Mnt/sand composite.

SEM micrographs of Mnt and Mnt/sand are presented in Fig. 2a and b, respectively. The pure Mnt sample showed small grains with a uniform texture and minimal agglomeration. On the other hand, the Mnt/sand samples had a granular morphology with layers and pores on their surfaces. The layer and porosity of the Mnt/sand sample increase the surface area available for adsorption. The pore, crack, and layered nature of the Mnt/sand sample help diffuse the PHY molecules toward the Mnt zones and layers.^33,35^ These structural features indicate that the Mnt/sand composite has suitable surface characteristics for the adsorption of organic pollutants from water.

Complementary BET specific surface area measurements were conducted to further evaluate the textural properties of the prepared materials. The specific surface areas of sand, Mnt, and the Mnt/sand composite were found to be 4, 230, and 45 m^2^ g^−1^, respectively. The very low surface area of sand confirms its limited direct contribution to PHY adsorption and supports its role mainly as a mechanically stable and permeable support. In contrast, Mnt exhibited a much higher surface area, consistent with its layered clay structure and active role in adsorption. The Mnt/sand composite showed an intermediate surface area of 45 m^2^ g^−1^, which is consistent with the incorporation of 20 wt% Mnt into the sand matrix. This result confirms that the composite retained a significant accessible surface area from the Mnt phase while gaining the operational advantages of the sand support.

**Fig. 3:**
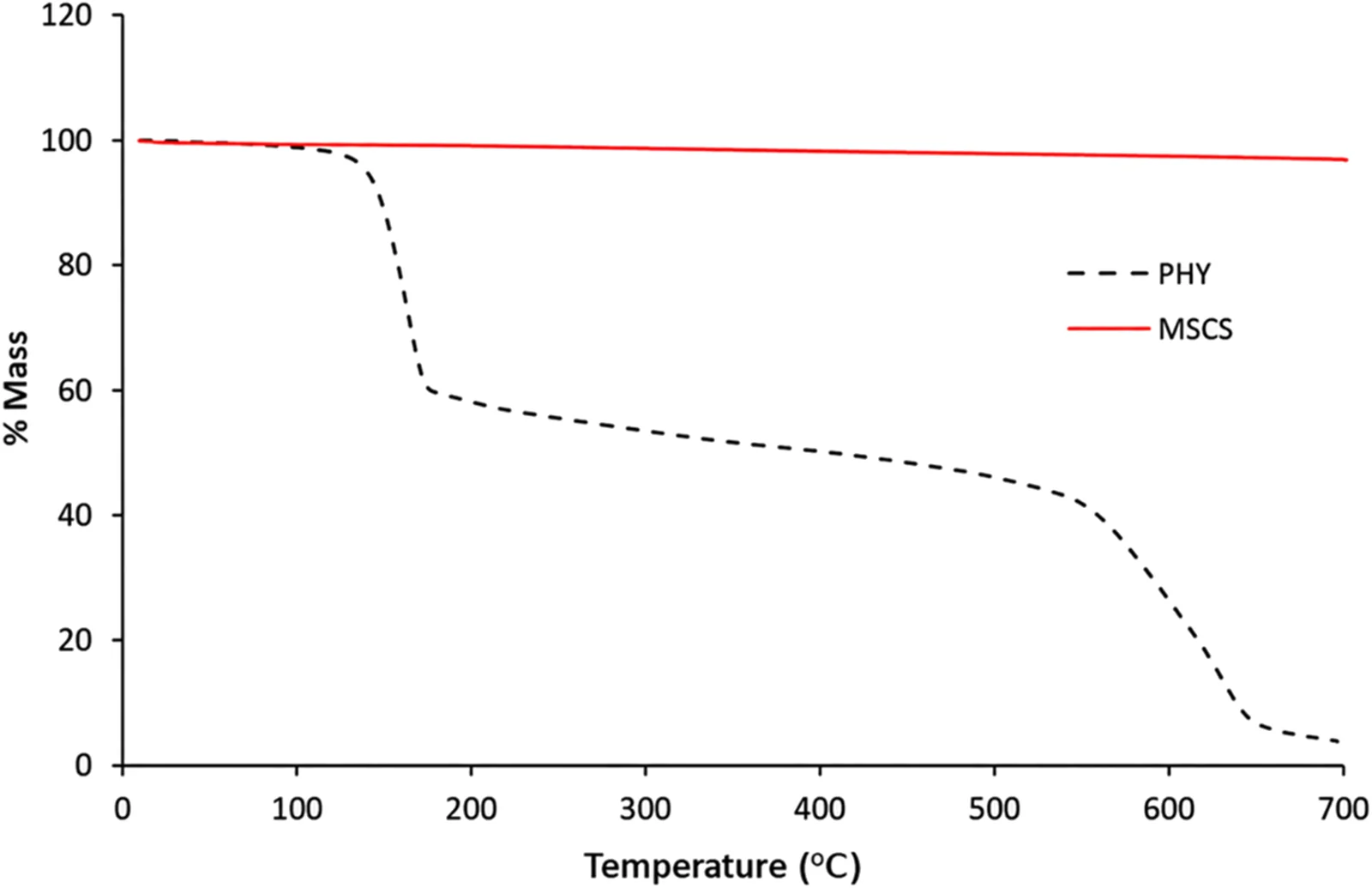
TGA curves of PHY and the Mnt/sand composite (MSCS). The mass profiles were normalized to the initial sample mass at the beginning of the measurement.

The thermal stability of the prepared Mnt/sand composite was evaluated by thermogravimetric analysis (TGA), as shown in Fig. 3. The mass profiles were normalized to the initial sample mass at the beginning of the measurement. The Mnt/sand composite (MSCS) exhibited only a minor mass loss over the investigated temperature range, with the residual mass decreasing gradually from approximately 100 wt% to approximately 97 wt% at 700 °C. This negligible weight loss indicates the high thermal stability of the inorganic composite and confirms its suitability for thermal regeneration at the selected operating temperature. In contrast, free PHY showed a multistep thermal decomposition profile. A pronounced mass loss occurred at approximately 150–180 °C, followed by progressive decomposition up to approximately 550 °C and near-complete degradation by approximately 650–700 °C. These findings demonstrate that the Mnt/sand composite remains structurally stable under thermal treatment conditions, whereas PHY undergoes extensive decomposition within the same temperature range. Therefore, the TGA results support the feasibility of using the Mnt/sand composite as a thermally regenerable adsorbent for repeated adsorption–thermolysis cycles.

Overall, the XRD, SEM, FT-IR, and TGA results provided complementary information on the phase composition, surface morphology, functional groups, PHY adsorption, thermal regeneration, and thermal stability of the Mnt/sand composite. XRD confirmed the coexistence of the characteristic crystalline phases of Mnt and sand, SEM revealed the granular and heterogeneous surface morphology of the composite, FT-IR provided evidence of PHY interaction with the adsorbent and the removal of PHY-related residues following regeneration, and TGA demonstrated the thermal stability of the composite under the selected regeneration conditions. Collectively, these findings support the successful preparation of a thermally stable Mnt/sand adsorbent suitable for repeated adsorption–regeneration cycles.

### 3.2 Adsorption of PHY in a batch system

#### 3.2.1 Effects of the initial concentration and contact time

**Fig. 4:**
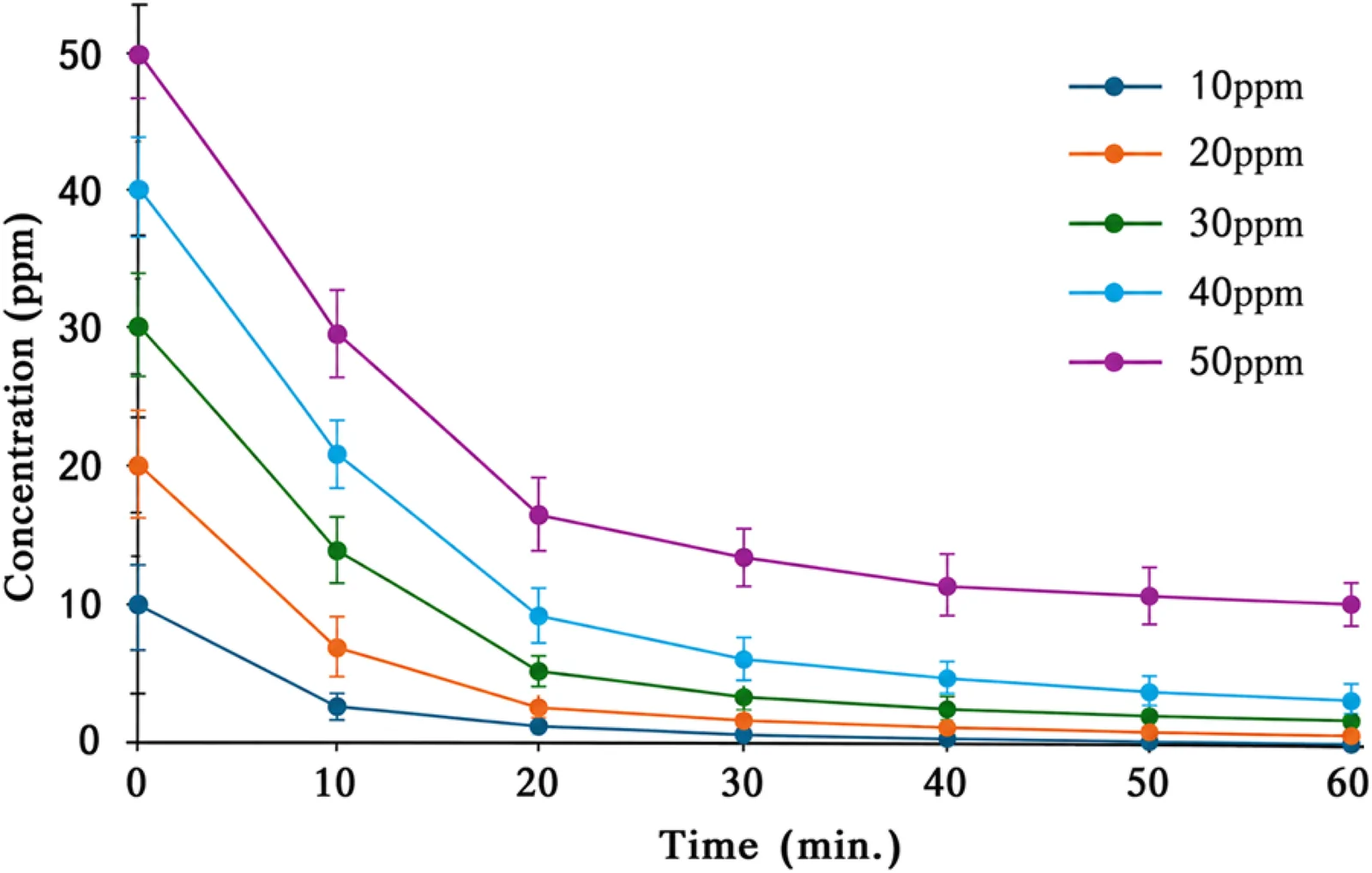
Profiles of phenazopyridine (PHY) concentration as a function of contact time during adsorption onto the Mnt/sand composite. Experimental conditions: initial concentration = 10–50 mg L^−1^, adsorbent dosage = 5 g L^−1^, solution pH = 5, volume = 100 mL, and temperature = 25 °C.

The effect of the starting concentration of PHY on the adsorption process of the Mnt/sand composite was studied at concentrations ranging from 10 to 50 mg L^−1^. As shown in Fig. 4, the adsorption process occurred rather quickly during the first stage of the process, with a significant reduction in the concentration of PHY within the first 20 minutes. Such rapid adsorption occurs because of the abundance of adsorption sites at the start of the process. Afterward, the adsorption rate starts to decrease because of the decrease in the number of free sites and the reduction in the concentration gradient. Equilibrium was achieved within approximately 60 minutes; thus, a contact time of 1 hour was used for further batch experiments. The removal efficiency decreased with increasing initial concentration from approximately 99% at 10 mg L^−1^ to approximately 80% at 50 mg L^−1^, whereas the adsorption capacity increased because of the greater number of adsorbed PHY molecules. At higher concentrations, the fixed number of adsorption sites becomes insufficient for the number of dissolved PHY molecules.

**Fig. 5:**
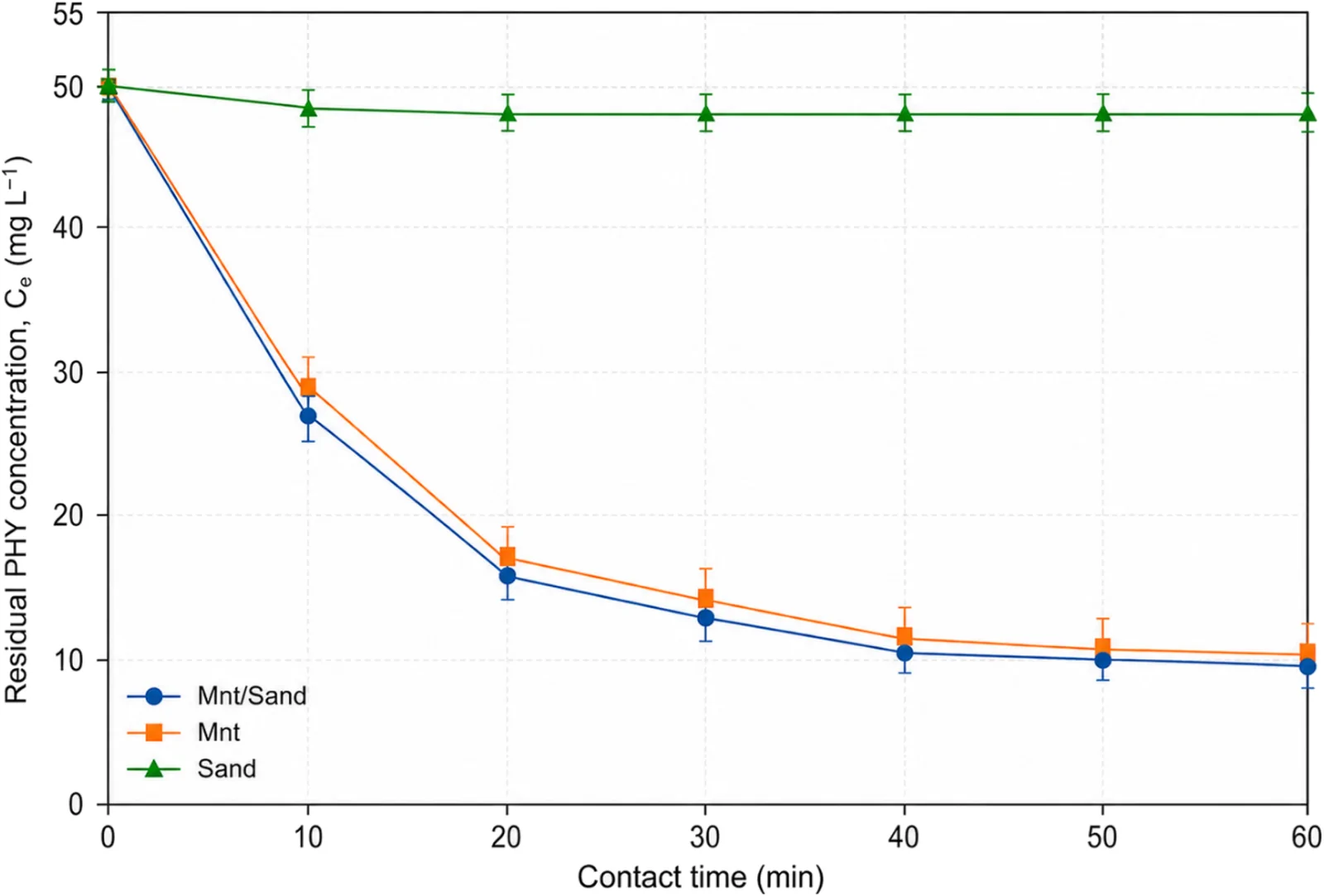
Comparison of residual PHY concentration profiles for Mnt/sand composite, Mnt alone, and sand alone at an initial PHY concentration of 50 mg L^−1^.

To explain the contribution of each part of the composite and to obtain a correct interpretation of the adsorption capacity, some control adsorption tests have been performed separately by using pure Mnt, pure sand and Mnt/sand composite under the same conditions. Adsorption tests were carried out in 100 mL of 50 mg L^−1^ PHY solution. Pure Mnt was 0.10 g, 0.40 g of pure sand was used, and 0.50 g of the Mnt/sand composite was used (0.10 g of Mnt and 0.40 g of sand). It is obvious from Fig. 5 that there is a rapid reduction in the residual PHY concentration in the case of Mnt and Mnt/sand, whereas sand alone results in negligible adsorption. The residual PHY concentrations after 60 min are 10.56, 48.70 and 10.39 mg L^−1^ for Mnt, sand and Mnt/sand, respectively. This corresponds to removal efficiencies of 78.88%, 2.60% and 79.22%, respectively (Table 1). Negligible adsorption was obtained in the case of sand, whose experimental adsorption capacity was 0.33 mg g^−1^. However, the Mnt/sand composite has an adsorption capacity of 7.92 mg g^−1^ on the basis of the total composite weight. When this value was normalized to the actual Mnt content in the composite, the capacity increased to 39.61 mg g^−1^, which is nearly identical to that of pure Mnt alone (39.44 mg g^−1^). Therefore, the relatively low apparent capacity of the Mnt/sand composite mainly results from dilution by the sand support rather than weak adsorption by Mnt. These results confirm that Mnt is the active phase responsible for PHY removal, whereas sand mainly serves as a mechanically stable support that improves adsorbent handling, separation, permeability, and suitability for fixed-bed operation.

**Table 1:** Control adsorption experiments comparing PHY removal by Mnt/sand composite, Mnt alone, and sand alone under identical conditions

Adsorbent	Composition/mass used	Final *C*_e_ (mg L^−1^)	PHY removal (%)	*q*_e_ Based on total adsorbent mass (mg g^−1^)	*q*_e_ Normalized to Mnt content (mg g^−1^)	Main conclusion
Mnt/sand composite	0.50 g composite = 0.10 g Mnt + 0.40 g sand	10.39	79.22	7.92	39.61	High removal; apparent capacity is diluted by sand mass
Mnt alone	0.10 g Mnt	10.56	78.88	39.44	39.44	Active adsorption phase responsible for PHY removal
Sand alone	0.40 g sand	48.70	2.60	0.33	—	Negligible adsorption; acts mainly as support

#### 3.2.2 Adsorption isotherms

Adsorption equilibrium data were analyzed by applying both the Langmuir and Freundlich isotherm models to gain further insight into the adsorption mechanism (Table S1). The Langmuir model is based on the assumption that adsorption occurs in a single layer on a homogeneous surface containing identical adsorption sites, whereas the Freundlich model describes adsorption on heterogeneous surfaces. The experimental data were fitted more accurately by the Langmuir model than by the Freundlich model, as indicated by the higher correlation coefficient (*R*^2^ = 0.996; Fig. S1). The Langmuir maximum adsorption capacity (*q*_max_) was 8.98 mg g^−1^ when calculated on the basis of the total mass of the Mnt/sand composite, and the Langmuir constant (*K*_L_) was 0.291 L mg^−1^. The Freundlich parameters also indicated favorable adsorption, with *n* = 2.216 and *K*_F_ = 2.304 (mg g^−1^) (L mg^−1^)^1/*n*^ (Table 2). However, because the composite contains a large fraction of sand support, the adsorption capacity must be interpreted carefully compared with that of clay-based adsorbents reported in the literature.

**Table 2:** Parameters of the Langmuir and Freundlich isotherm models

Isotherm	Langmuir			Freundlich		
Parameter	*q*_max_ (mg g^−1^)	*K*_L_ (L mg^−1^)	*R* ^2^	*K*_f_ ((mg g^−1^) (L mg^−1^))^1/*n*^	*n*	*R* ^2^
Value	8.98	0.291	0.996	2.304	2.216	0.925

Although the total mass-based Langmuir capacity of the Mnt/sand composite is lower than those reported for some clay-based adsorbents, such a direct comparison should be interpreted cautiously because many reported adsorbents are composed predominantly of active clay or chemically modified adsorption phases.^26,32–35^ The current composite consists of 20 wt% adsorbed Mnt and 80 wt% sand, which was intentionally introduced as a mechanically durable and porous substrate. Hence, the calculated *q*_max_ value of 8.98 mg g^−1^ is related to the total mass of the composite and includes both adsorbing and nonadsorbing sand. Normalizing the results for the Langmuir adsorption capacity for the actual content of Mnt gives 44.90 mg g^−1^ Mnt, thus offering a better point of comparison to the capacity values for clay adsorbents. This means that the low value of the total mass-based adsorption capacity is mostly due to dilution of the active phase with sand rather than the low adsorption capacity of Mnt. In other words, Mnt/sand composite must be evaluated not only on the basis of the mass adsorption capacity of the composite but also on the basis of its additional benefits, such as easy adsorbent recoverability, minimal losses, high permeability, low clogging tendencies, the possibility of use in fixed beds, and simple regeneration.

#### 3.2.3 Adsorption thermodynamics

Thermodynamics calculations were performed to investigate the feasibility and characteristics of the process of the adsorption of PHY by the Mnt/sand adsorbent. Thermodynamic constants such as the standard Gibbs free energy (Δ*G*°), standard enthalpy (Δ*H*), and standard entropy change (Δ*S*°) were determined.

Adsorption experiments were performed at different temperatures ranging from 283 to 323 K (10–50 °C) to determine the temperature dependence of the adsorption process. The values of Δ*H*° and Δ*S*° were obtained from the slope and intercept of the linear plot of ln *K*_d_*versus* 1/*T*, as presented in Fig. S2. The calculated thermodynamic parameters are summarized in Table 3.

**Table 3:** Thermodynamic parameters of PHY adsorption on Mnt/sand

Parameter	Δ*G*° (kJ mol^−1^)					Δ*H*° (kJ mol^−1^)	Δ*S*° (J mol^−1^ K^−1^)
Values	283 K	293 K	303 K	313 K	323 K	18.253	55.784
	−0.733	−2.463	−3.192	−3.680	−3.444		

The positive value of the standard enthalpy change (Δ*H*° = 18.253 kJ mol^−1^) indicates that the adsorption of PHY onto the Mnt/sand adsorbent is an endothermic process. The above findings indicate that higher temperatures favor the adsorption ability, which is in line with the experimental findings acquired from temperature-dependent adsorption studies.

The Gibbs free energy change values (Δ*G*°) are negative for all the temperatures studied, and the values range between −0.733 kJ mol^−1^ and −3.680 kJ mol^−1^. The negative values of Δ*G*° indicate that the adsorption reaction is spontaneous. Moreover, the value of Δ*G*° becomes more negative with increasing temperature, indicating the improved thermodynamic feasibility of the adsorption reaction.

The increase in entropy (Δ*S*° = 55.784 J mol^−1^ K^−1^) represents increased disorder at the solid-solution interface when adsorption takes place. This may be due to the displacement of the structured water molecules that have been previously associated with the surface of the adsorbent and the PHY molecules into the bulk solution. Thus, the degrees of freedom of the system increase, which contributes to the increase in entropy.

Moreover, the increase in entropy is highly important for the thermodynamic feasibility of the adsorption process at high temperatures. On the basis of the Gibbs energy equation (Δ*G*° = Δ*H*° − *T*Δ*S*°), the increase in entropy is particularly important when the temperature is increased. As a result, the *T*Δ*S*° term reduces the positive contribution of the enthalpy to the Gibbs energy. Thus, more negative Gibbs energies are formed at higher temperatures.

#### 3.2.4 Adsorption kinetics

Adsorption kinetics were investigated to better understand the rate and mechanism of PHY adsorption onto the Mnt/sand composite. Kinetic analysis provides valuable insight into the adsorption mechanism and the potential rate-controlling steps governing the interaction between the adsorbate molecules and the adsorbent surface. The experimental data were evaluated using three widely applied kinetic models: the pseudo-first-order, pseudo-second-order, and intraparticle diffusion models. The mathematical formulations of these models are provided in the S1 (Fig. S3).

The linear plots obtained from the kinetic analyses are shown in Fig. S3, including the pseudo-first-order model (Fig. S3a), a pseudo-second-order model (Fig. S3b), and an intraparticle diffusion model (Fig. S3c). The corresponding kinetic parameters derived from these models are summarized in Table 4.

**Table 4:** Parameters of the pseudofirst-order model, pseudosecond-order model, and intraparticle diffusion model

The model	Pseudo first order kinetic model			Pseudosecond order kinetic model			Intraparticle diffusion kinetic model		
Parameters of the model	*K*_1_ (min^−1^)	*q*_e_ (mg g^−1^)	*R* ^2^	*K*_2_ (g mg^−1^ min^−1^)	*q*_e_ (mg g^−1^)	*R* ^2^	*K*_*i*_ (mg g^−1^ min^−1/2^)	*C*	*R* ^2^
Values	0.089	1.139	0.946	0.347	2.191	0.997	0.511	2.773	0.756

The pseudo-second-order model provided the best fit to the experimental kinetic data, as indicated by the high correlation coefficient (*R*^2^ = 0.997) and the close agreement between the calculated and experimental *q*_e_ values. Although the pseudo-second-order model is frequently associated with adsorption systems involving significant surface interactions, kinetic model fitting alone cannot be considered definitive evidence of a purely chemical adsorption mechanism. Rather, the excellent fit of the pseudo-second-order model suggests that surface adsorption processes play an important role in controlling the adsorption rate of PHY onto the Mnt/sand composite.

The Weber–Morris intraparticle diffusion plot did not pass through the origin and exhibited a significant intercept (*C* = 2.773), indicating that intraparticle diffusion was not the sole rate-controlling step in the adsorption process. These results suggest that PHY adsorption onto the Mnt/sand composite involved multiple mechanisms, including boundary-layer diffusion, intraparticle diffusion, and surface adsorption processes.

The activation energy of the adsorption was deduced from the Arrhenius plot of ln *k* against 1/*T* (Fig. S4). The activation energy of adsorption obtained from the Arrhenius plot was 30.4 kJ mol^−1^. This value lies within the intermediate energy region commonly associated with mixed adsorption behavior involving both physical interactions and stronger surface interactions. Therefore, the adsorption of PHY onto the Mnt/sand composite is more appropriately described as a multimechanistic process rather than a purely chemisorption-controlled system. The excellent agreement with the pseudo-second-order model likely reflects the significant contribution of surface adsorption interactions to the adsorption kinetics without necessarily indicating exclusive chemical adsorption. Although additional residual error analysis may provide further statistical comparisons among kinetic models, the present interpretation was based primarily on correlation coefficients together with the physicochemical consistency of the adsorption system.

#### 3.2.5 Effects of pH and adsorbent dose

**Fig. 6:**
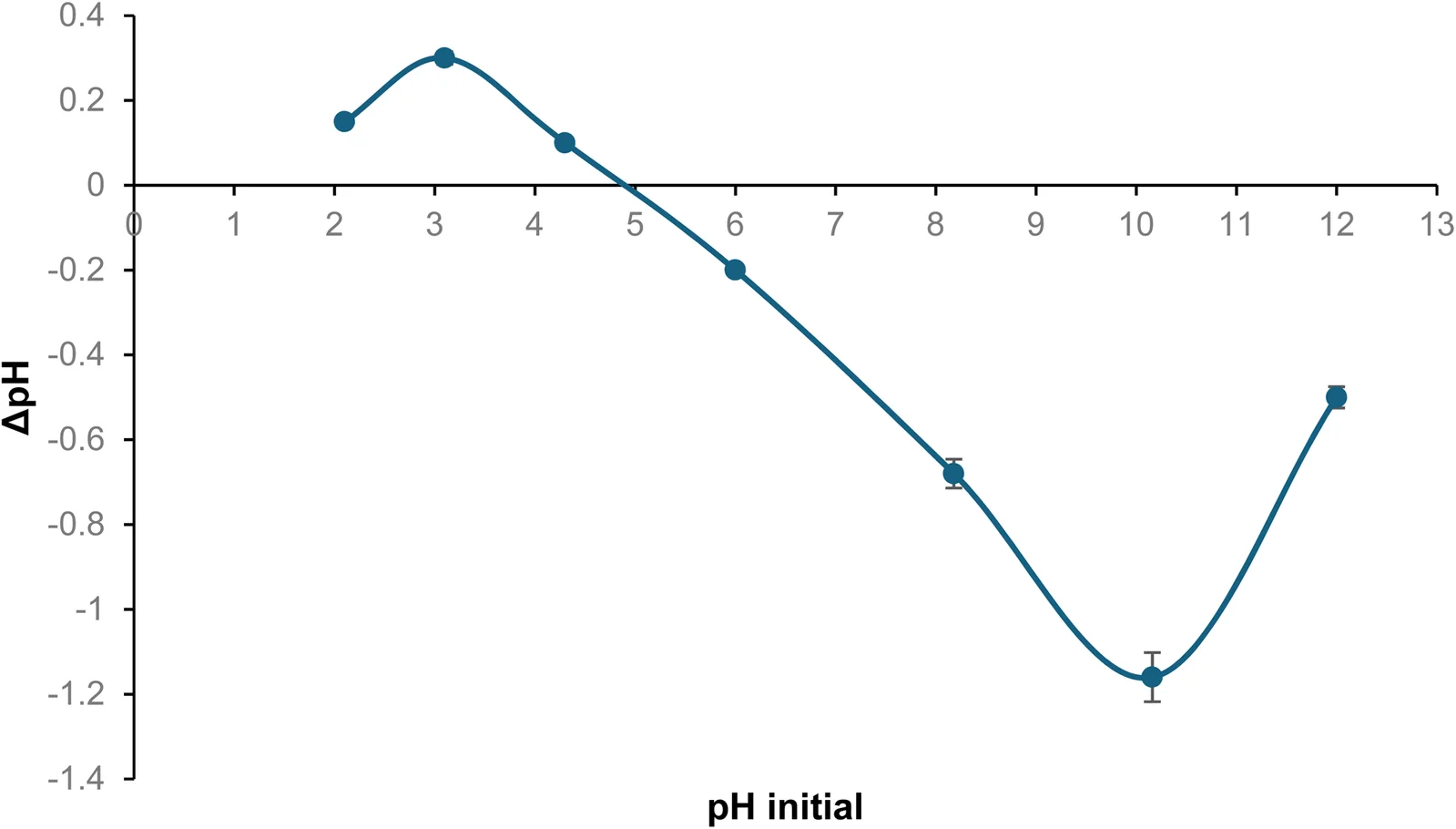
Plot of delta pH *versus* initial pH. The results were obtained at room temperature.

The pH of a solution strongly affects how PHY adsorbs on Mnt/sand composites, as it alters the surface charge of the adsorbent as well as the species of the adsorbate.^33,37^ The point of zero charge (pH_pzc_) of the composite was found to be approximately 4.8 by plotting the pH *vs.* the initial pH of the suspension (Fig. 6). This means that the adsorbent surface becomes positively charged at pH values less than 4. 8 and negatively charged at pH levels above it. Since PHY is a weak base with a p*K*_a_ of approximately 5.2, its protonation state changes with the solution pH.

**Fig. 7:**
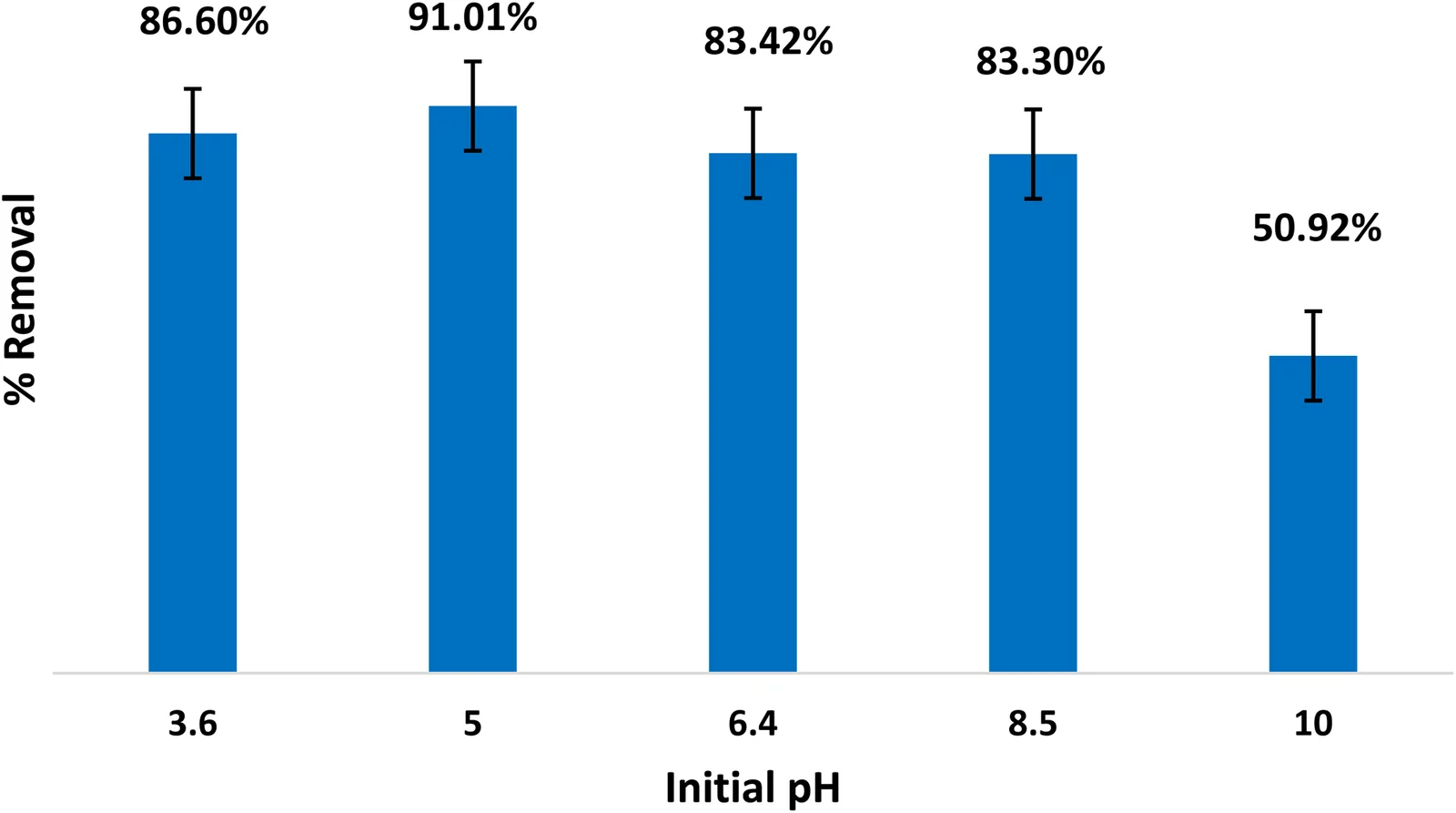
Effect of initial solution pH on the percentage removal of phenazopyridine (PHY) by the Mnt/sand composite. Experimental conditions: initial concentration = 30 mg L^−1^, adsorbent dosage = 5 g L^−1^, solution volume = 100 mL, temperature = 25 °C.

The effect of pH on the adsorption efficiency is presented in Fig. 7. The maximum removal was observed at pH 5, where the Mnt/sand surface is expected to be negatively charged because this pH is slightly above the pH_pzc_ of the composite, whereas PHY exists largely in its protonated cationic form because the pH is close to its p*K*_a_. Therefore, electrostatic attraction between protonated PHY and negatively charged surface sites is an important contributor to the enhanced adsorption at pH 5. However, the adsorption mechanism should not be attributed solely to electrostatic attraction. Noticeable PHY removal was still observed under acidic and near-neutral conditions, where electrostatic attraction is weakened or partially unfavorable, indicating the contribution of nonelectrostatic interactions. This interpretation is supported by FT-IR analysis, which revealed PHY-related bands on the contaminated Mnt/sand surface, and by DFT results showing close contacts between PHY functional groups and oxygen-rich Mnt surface sites. The optimized DFT geometry indicated hydrogen bond-type interactions involving the amino groups of PHY and siloxane oxygen atoms of Mnt, together with additional contacts involving the azo group. Additionally, the negative adsorption energy computed (−334.25 kJ mol^−1^, −3.46 eV) further supports the energetically favorable interaction between PHY and Mnt. Therefore, the increased adsorption at pH 5 could be better explained by a combination of interactions, such as electrostatic interactions, hydrogen bonding, van der Waals interactions, and surface polarization, instead of electrostatic interactions only.

When the pH approaches neutrality (*i.e.*, approximately 6.5), the PHY compound is found to be mostly present in its deprotonated and neutral state, thus leading to a reduction in the adsorption efficiency. At high alkalinity levels (*i.e.*, a pH of approximately 10), there was also a reduction in the adsorption efficiency because of the presence of more hydroxyl ions and electrostatic repulsion between the negatively charged molecules and adsorbent.

Under strongly acidic conditions (pH ∼3.6), where the solution pH is below the pH_pzc_, the adsorbent surface becomes positively charged. Under these conditions, electrostatic repulsion between protonated PHY molecules and the positively charged adsorbent surface may contribute to the observed decrease in adsorption efficiency. Nevertheless, some degree of adsorption still occurred, suggesting the contribution of additional nonelectrostatic interactions and possible adsorption within the porous structure of the composite.

Although zeta potential measurements at different pH values were not performed in the present study, the adsorption behavior observed near the pH_pzc_ and p*K*_a_ values supports the contribution of both electrostatic and nonelectrostatic adsorption mechanisms. In addition to the pH, the amount of adsorbent also affects the removal efficiency of PHY from aqueous solution. Increasing the Mnt/sand dosage increased the overall PHY removal efficiency because of the greater availability of adsorption sites. However, the adsorption capacity per unit mass of adsorbent decreased when higher dosages were used, probably because with decreasing pollutant concentration, only some active sites were occupied, leading to the underutilization of the remaining sites.

#### 3.2.6 Effect of adsorbent dose

**Fig. 8:**
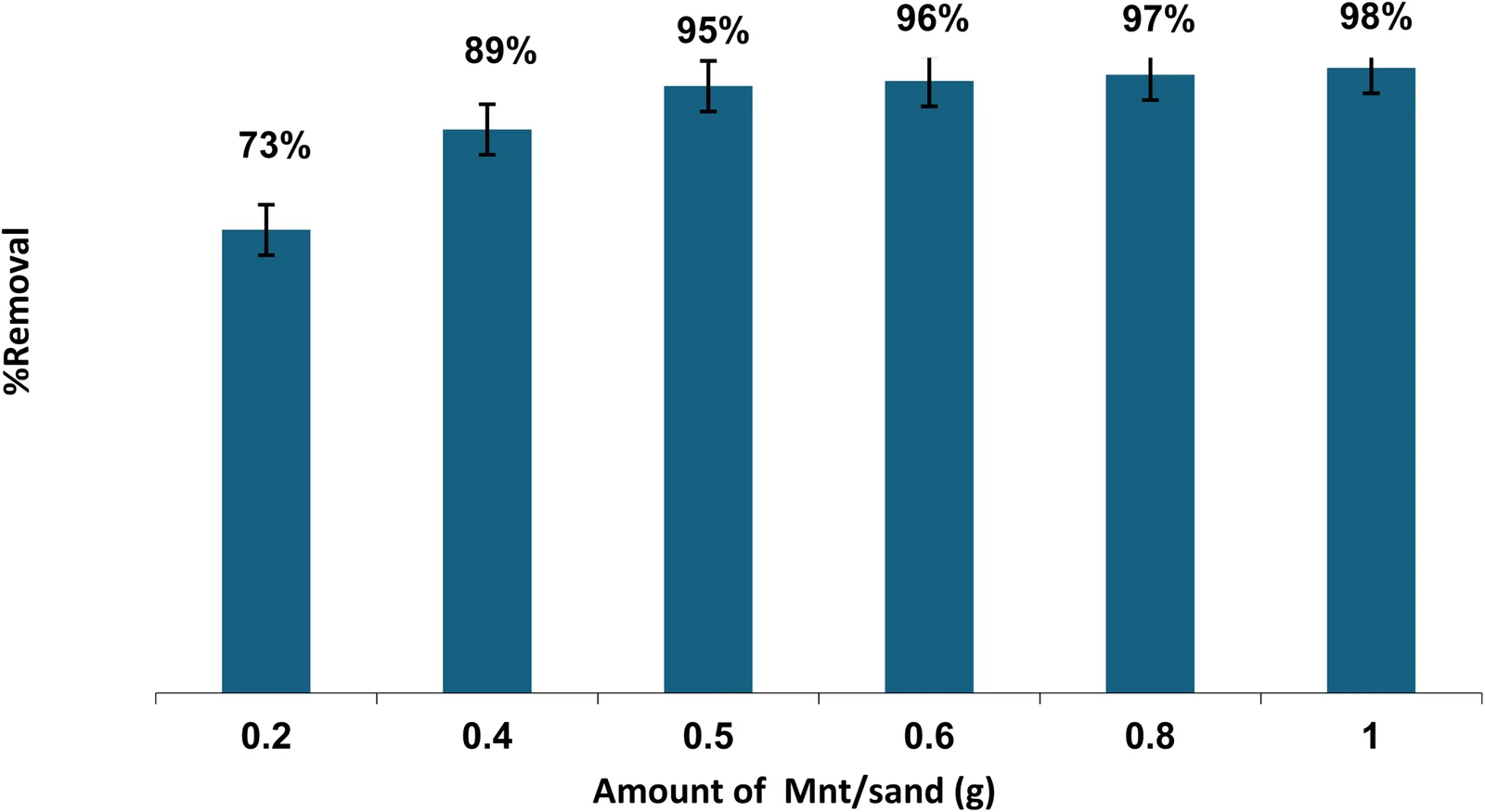
Effect of Mnt/sand dosage on the removal of phenazopyridine (PHY). Experimental conditions: initial concentration = 30 mg L^−1^, solution pH = 5, volume = 100 mL, temperature = 25 °C.

The influence of the adsorbent dosage on the adsorption of PHY by the Mnt/sand composite was studied by changing the adsorbent quantity and keeping the other conditions fixed. The correlation of the adsorbent amount with the percentage removal of PHY and the adsorption capacity (*q*_e_) is shown in Fig. 8. The percentage removal of PHY increases as the Mnt/sand dosage increases. This is because more adsorption sites become available, and there is an increase in the total surface area of the adsorbent, which leads to better interaction of the adsorbent with pollutant molecules.

**Fig. 9:**
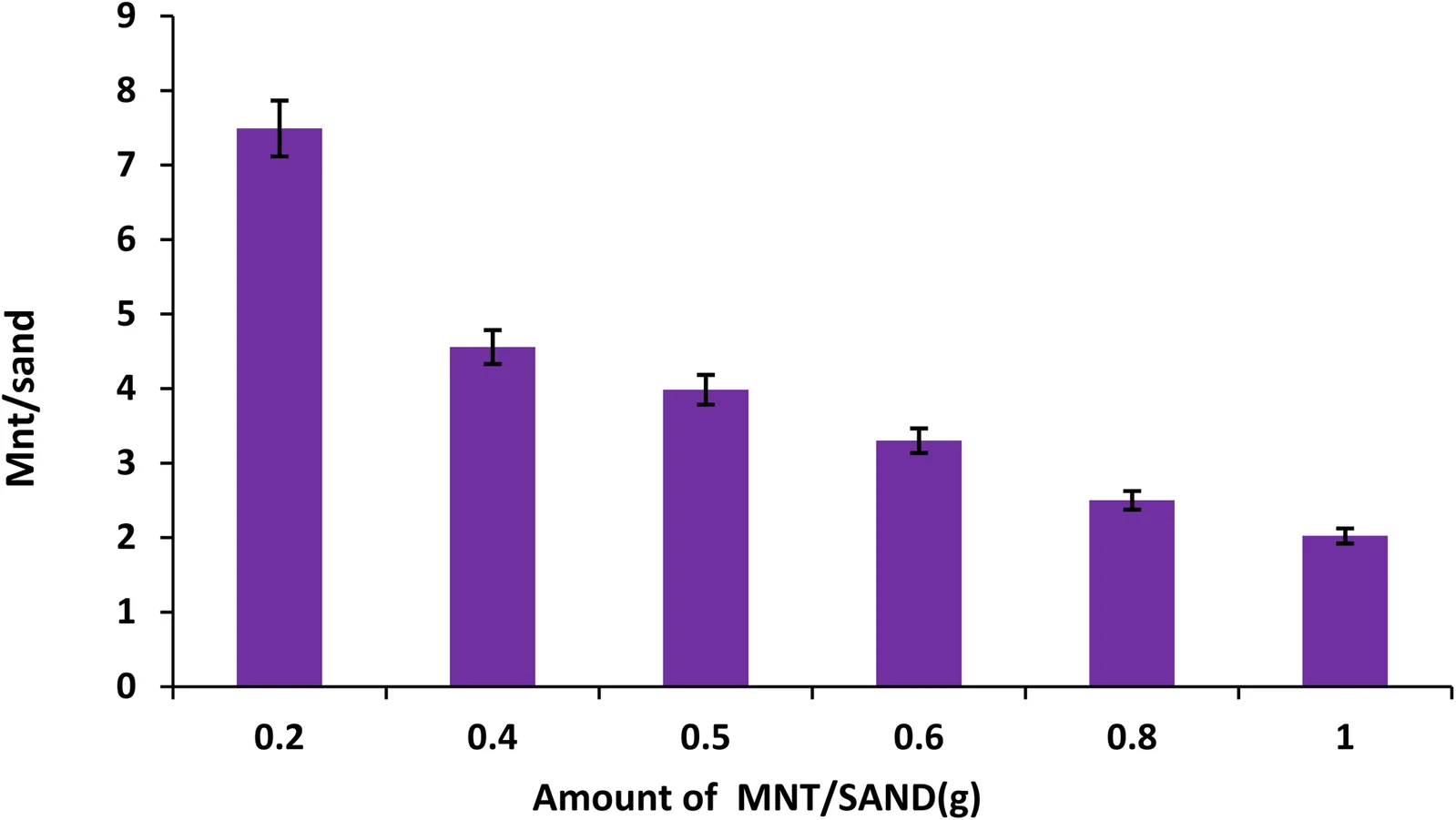
Effect of Mnt/sand dosage on the adsorption capacity of phenazopyridine (PHY). Experimental conditions: initial concentration = 30 mg L^−1^, solution pH = 5, volume = 100 mL, temperature = 25 °C.

However, as shown in Fig. 9, the adsorption capacity per unit mass of adsorbent (*q*_e_) decreases as the adsorbent dosage increases. The reason behind this decrease is that the concentration of the available PHY becomes too low to fully saturate all the adsorption sites when the amount of adsorbent is increased. Consequently, a number of the adsorption sites are left unutilized, which results in a decrease in the adsorption capacity per unit mass. In addition, increasing the adsorbent dosage accelerates the adsorption rate because of the greater availability of active sites. On the basis of these results, an adsorbent dosage of 0.5 g was identified as the optimal amount for the adsorption process, since further increases in adsorbent mass mainly increase the adsorption rate without significantly improving the overall removal efficiency.

#### 3.2.7 Regeneration and reuse of the adsorbent

**Fig. 10:**
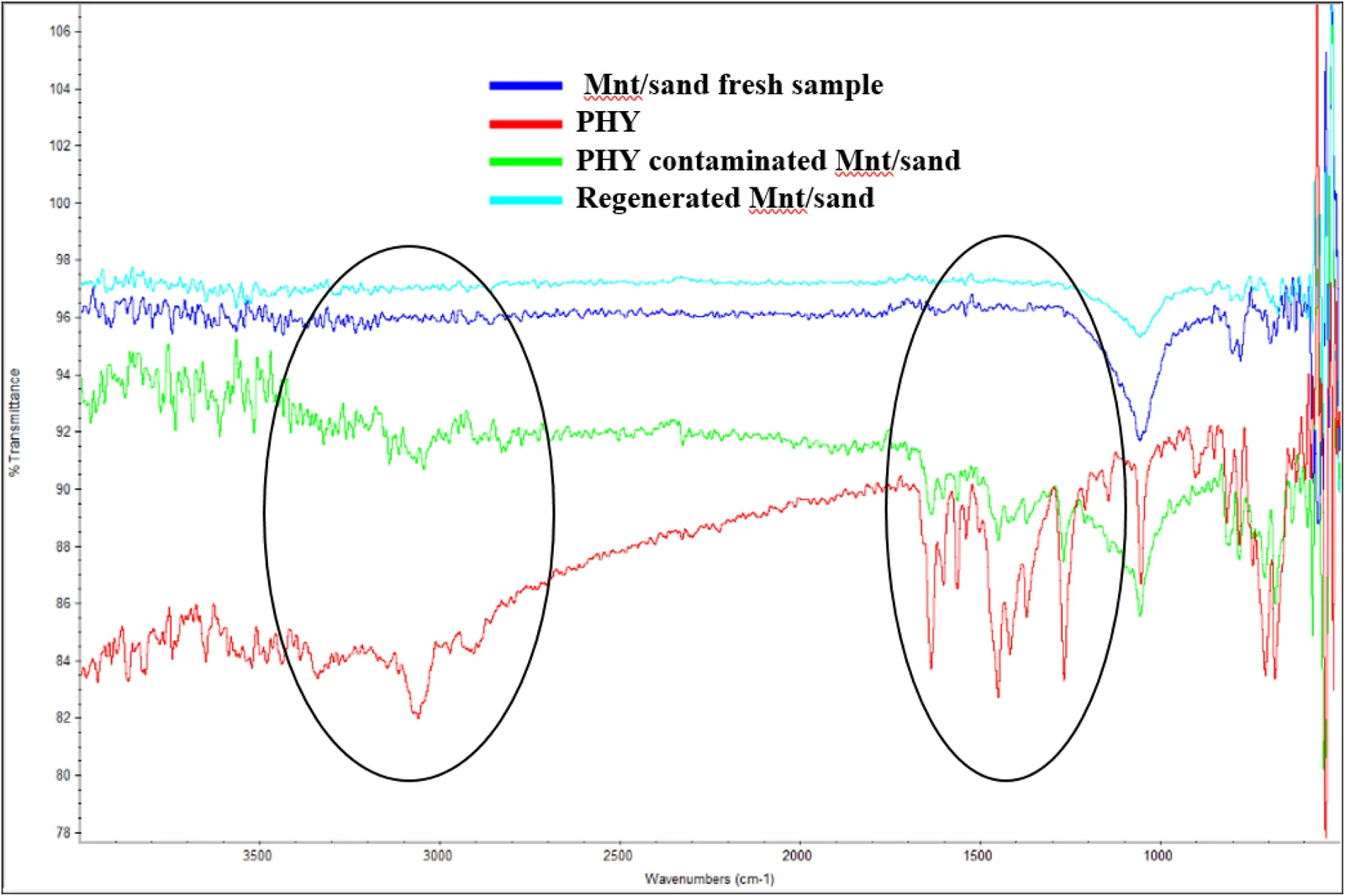
FT-IR spectra of fresh Mnt/sand, PHY, PHY-contaminated Mnt/sand, and Mnt/sand regenerated at 550 °C for 2 h. The disappearance of PHY-related bands after thermal regeneration, particularly in the 1200–1640 cm^−1^ region, supports the effective decomposition/removal of adsorbed PHY residues. An enlarged inset is included to distinguish the fresh and regenerated Mnt/sand spectra.

The regeneration of the Mnt/sand adsorbent was achieved through thermal treatment (thermolysis) after the adsorption of PHY. FT-IR spectroscopy was used to investigate the interaction between PHY and the adsorbent surface and to evaluate the effectiveness of the regeneration process (Fig. 10). The FT-IR spectrum of fresh Mnt/sand shows characteristic bands corresponding to the structural components of the composite, including a strong band at approximately 1050 cm^−1^ attributed to Si–O stretching vibrations associated with silanol groups and silica structures. Additional broad bands observed near 3618 and 3448 cm^−1^ correspond to the –OH stretching vibrations of structural hydroxyl groups and adsorbed water molecules within the Mnt layers. Other bands at approximately 650 and 620 cm^−1^ are assigned to the vibrations of Si–O–Al and Si–O–Mg, respectively.^40^ On the other hand, the band near 804 cm^−1^ is due to quartz.^45^ The FT-IR spectrum of PHY displays significant peaks that can be attributed to N–H stretching vibrations, aromatic C–H stretching, and azo groups (N=N). With respect to PHY-contaminated Mnt/sand, the FT-IR spectrum shows additional PHY-related bands mainly in the 1200–1640 cm^−1^ region, confirming the adsorption of PHY molecules on the composite surface.^33,46^ After thermal regeneration at 550 °C for 2 h, these PHY-related bands disappeared, and the spectrum of the regenerated Mnt/sand closely resembled that of the fresh composite. These results provide experimental evidence that the adsorbed PHY residues were effectively decomposed or removed from the adsorbent surface under the selected regeneration conditions. Importantly, the persistence of the characteristic Mnt/sand bands after regeneration indicates that the composite structure was not significantly altered by the thermal treatment.

The appearance of PHY-related bands after adsorption provides spectroscopic evidence for the association of PHY with the Mnt/sand surface. Since the 1200–1640 cm^−1^ region includes contributions from aromatic, nitrogen-containing, and azo-related vibrations, its presence in the PHY-contaminated adsorbent supports the involvement of PHY functional groups in surface binding. Together with the broad –OH bands and oxygen-rich siloxane framework of Mnt/sand, these spectral changes are consistent with the contributions of hydrogen bonding and other secondary interactions to PHY adsorption in addition to electrostatic attraction.

**Fig. 11:**
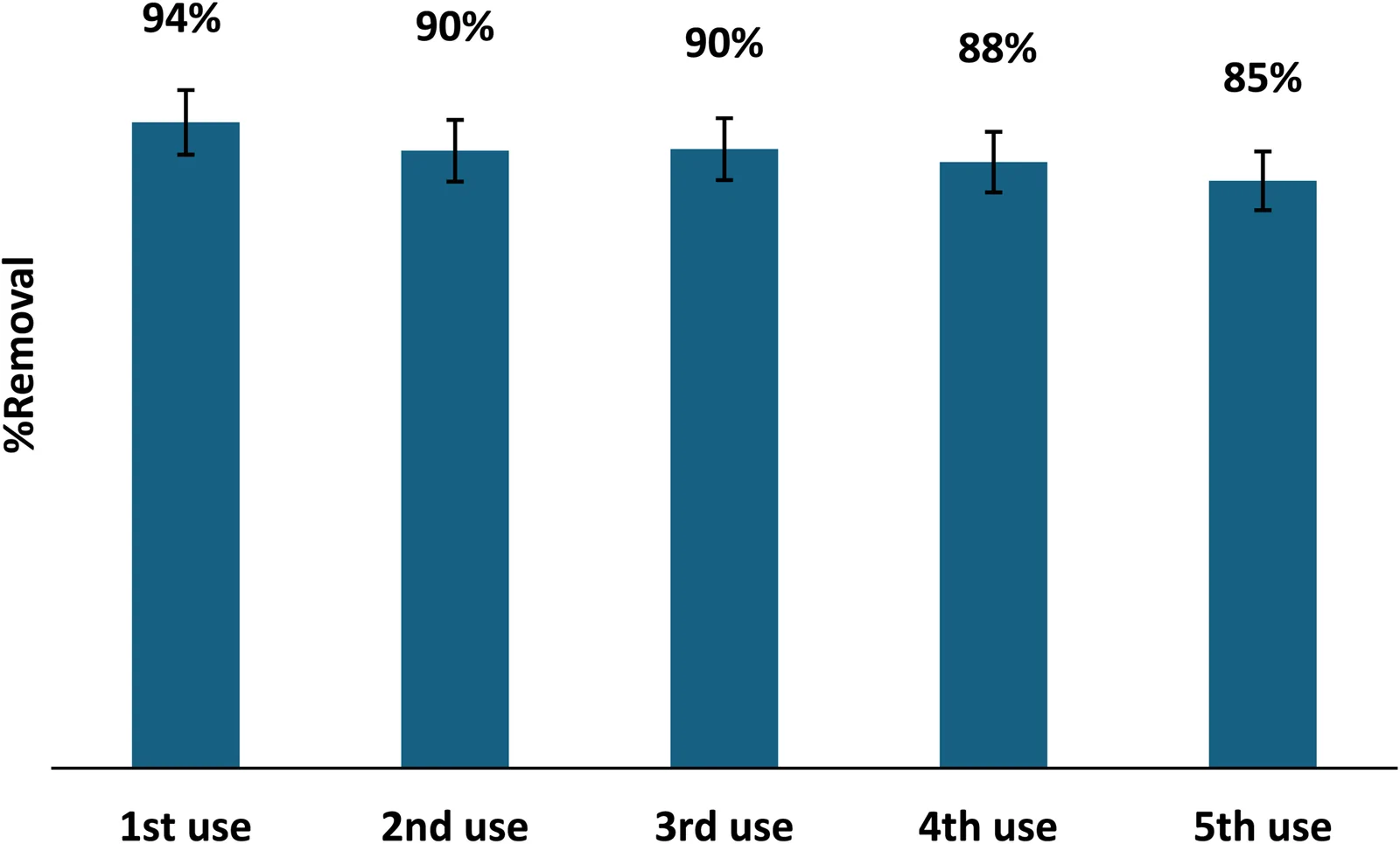
Removal efficiency of phenazopyridine (PHY) using Mnt/sand composite over five consecutive adsorption–regeneration cycles. Experimental conditions: initial concentration = 30 mg L^−1^, adsorbent dosage = 5 g L^−1^, solution pH = 5, volume = 100 mL, temperature = 25 °C.

The potential of the regenerated Mnt/sand adsorbent for repeated use was examined by conducting several adsorption cycles. A total of 0.5 g of the adsorbent was used for each time it was in contact with 100 mL of 20 ppm PHY at room temperature, and a 1 h contact time was used for each cycle. After the adsorption step, the adsorbent was regenerated by thermolysis at 550 °C for 2 h before being reused in the next adsorption cycle. The data illustrated in Fig. 11 indicate that the adsorption efficiency was still quite good after five successive cycles of adsorption, with PHY removal decreasing from approximately 94% in the first cycle to 85% after the fifth cycle. These findings indicate that the Mnt/sand composite has good structural stability and preserves its adsorption capacity after several regeneration cycles, which emphasizes thermolysis as an effective method of regeneration and, correspondingly, of this adsorbent in eco-friendly water treatments.

### 3.3 Adsorption of PHY in a continuous flow system

Batch adsorption experiments are quick and effective ways to determine adsorption performance at the bench level but usually involve several steps, such as mixing, centrifuging, and separating phases, which can be challenging and not ideal if scaling up is considered. In addition, one cannot be sure that the scale-up of batch systems could yield the same efficiency as that at the laboratory level. Hence, to test the practical potential of the developed adsorbent, the adsorptive removal of PHY was performed one step further in a continuous flow column system. The Mnt/sand composite was made in granular form to achieve proper permeability and mechanical stability in the column for efficient flow of the influent solution through the packed bed. Column adsorption was assessed by changing different process parameters, such as the initial PHY concentration, flow rate, column height, and solution temperature, with the aim of making the system suitable for large-scale water treatment applications. In all the column experiments, a total influent solution volume of 100 mL was passed through the packed bed under the selected flow conditions.

To improve the continuous-flow analysis while keeping the main text concise, a representative breakthrough profile was analyzed to obtain the main fixed-bed performance indicators, including breakthrough time, treated volume at breakthrough, breakthrough capacity, cumulative capacity, model-derived saturation capacity, and bed utilization efficiency. The detailed numerical dataset, calculation tables, and Thomas-model fitting are provided in SI Section S5.

#### 3.3.1 Effect of initial concentration

**Fig. 12:**
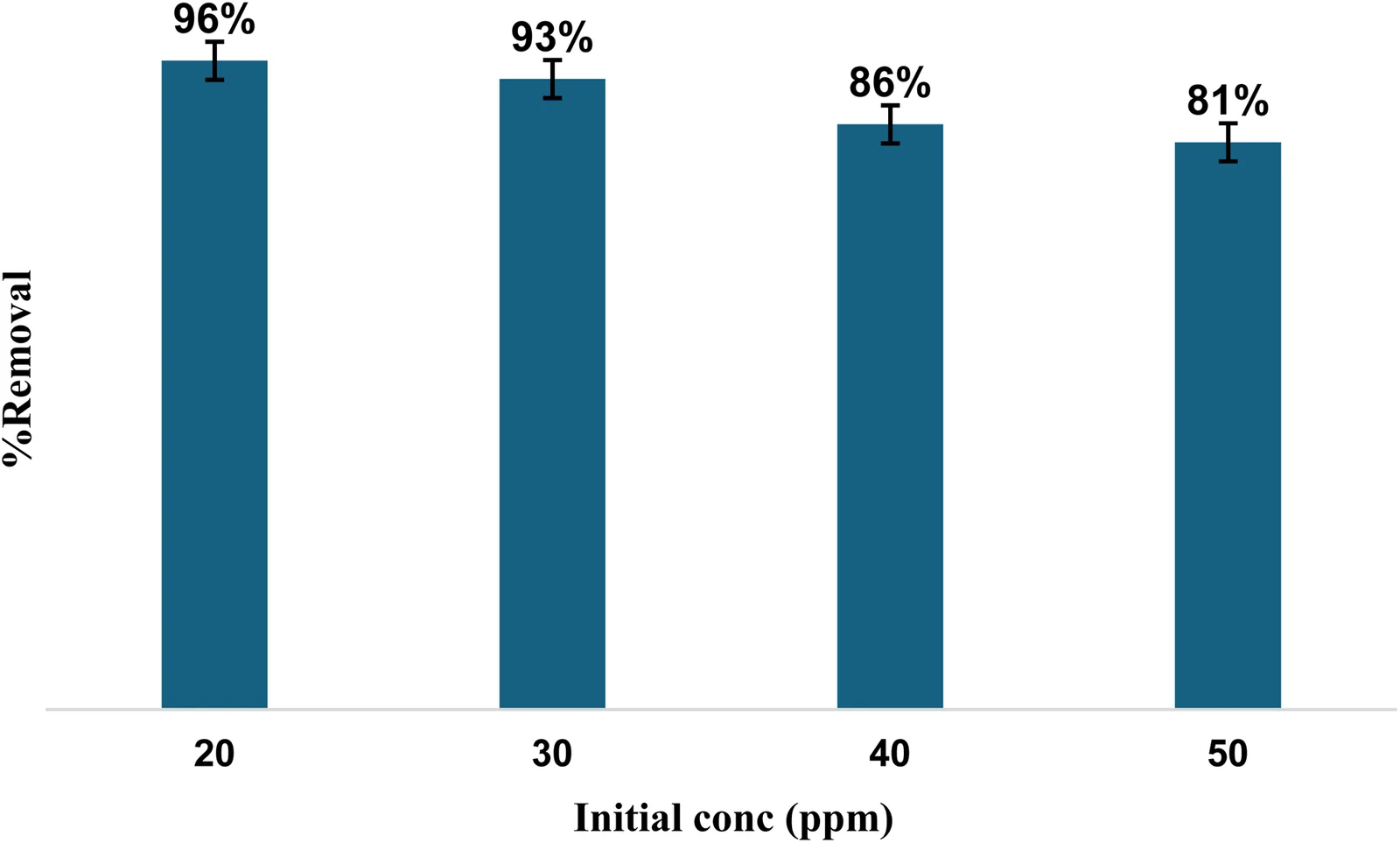
Effect of initial influent concentration of phenazopyridine (PHY) on removal efficiency in a continuous-flow system. Experimental conditions: flow rate = 10 mL min^−1^, bed adsorbent mass = 3.0 g, column height = 4 cm, and solution temperature = 25 °C.

The effect of the initial PHY concentration on the effectiveness of column adsorption was examined by changing the influent concentration from 20 to 50 ppm while the other operational parameters were kept unchanged (flow rate of 10 mL min^−1^, adsorbent mass of 3.0 g, solution volume of 100 mL, column height of 4 cm, and temperature of thermal regeneration of 550 °C) (Fig. 12). As shown in Table 5, the quantity of PHY adsorbed increased as the initial concentration changed because the greater number of pollutant molecules entering the column resulted in more interactions with the adsorption sites. In contrast, the total removal percentage decreased from 96% to 81% when the concentration increased. The reason for this behavior is that when their molecules leave a smaller portion of the active sites exposed and the quality of the effluent being reduced, the pollutant concentration levels increase, which is a greater portion of the column time.

**Table 5:** Effects of various parameters on the adsorption removal of PHY in the column system

Factors	*X*	Amount adsorbed	Removal efficiency (%)
Initial concentration (ppm)	20	1.91	96
	30	2.78	93
	40	3.44	86
	50	4.08	81
Flow rate (mL min^−1^)	2	4.85	97
	4	4.74	95
	6	4.29	85
	8	4.10	82
	10	3.97	80
Height of column (cm)	8	4.74	95
	4	4.32	86
Temperature of solution (°C)	10	4.50	90
	20	4.65	93
	30	4.75	95
	40	4.80	96
	50	4.85	97

#### 3.3.2 Effect of flow rate

**Fig. 13:**
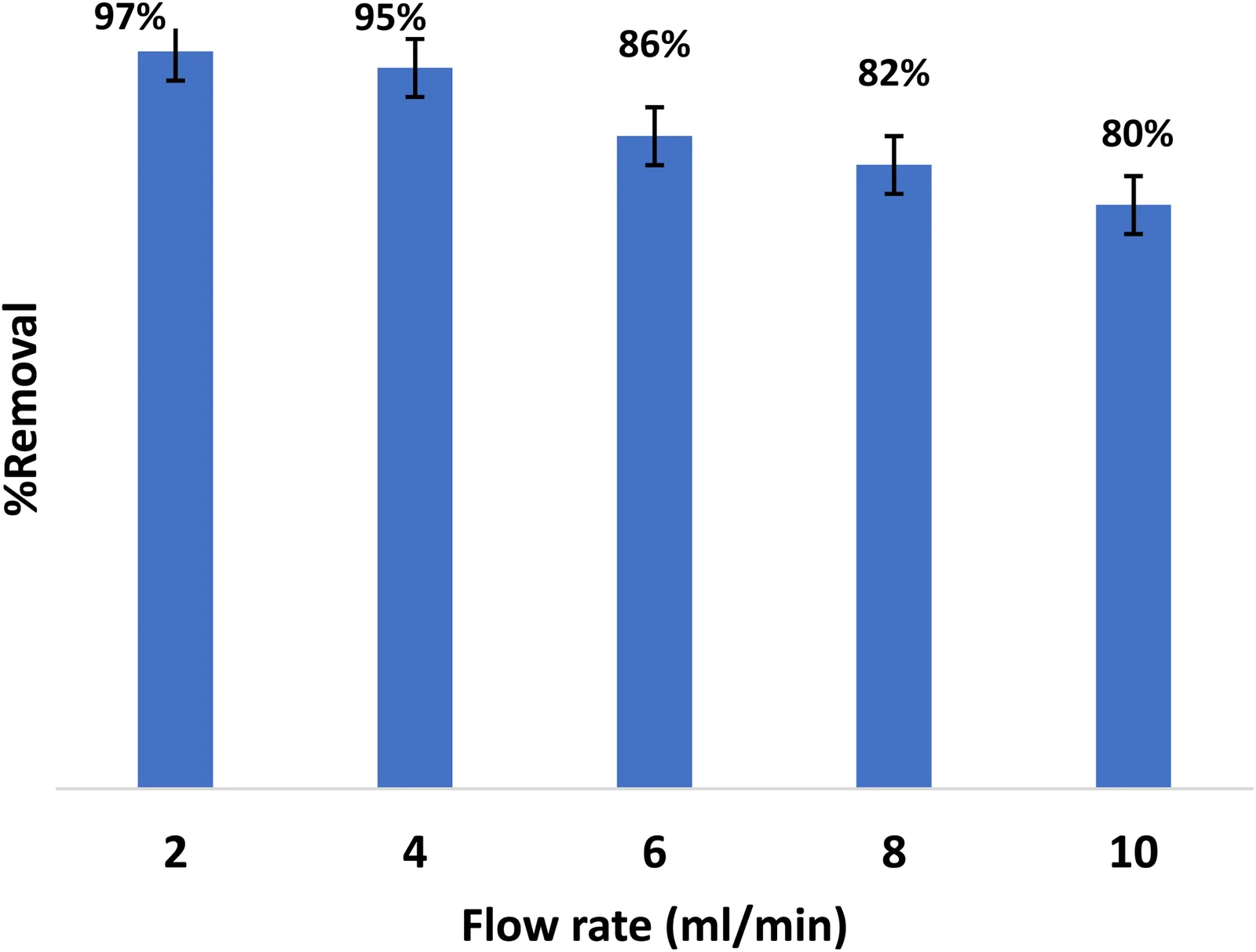
Breakthrough curves and overall removal efficiencies for PHY adsorption onto the Mnt/sand composite at different flow rates. Experimental conditions: initial PHY concentration = 50 mg L^−1^, adsorbent mass = 3 g, bed height = 8 cm, treated solution volume = 100 mL, and flow rates = 2–10 mL min^−1^.

The flow rate effect was also studied between 2 and 10 mL min^−1^ (Fig. 13). The results in Table 5 indicate that both the adsorption capacity and the removal efficiency decreased as the flow rate increased. When the flow rate is high, the time for the PHY solution to stay inside the column becomes very limited; thus, there is not enough time for the pollutant molecules to contact the adsorbent surface. Thus, adsorption equilibrium is not fully reached before the solution leaves the column. On the other hand, with lower flow rates, the contact times are longer, which means that the adsorbate and adsorbent materials can interact more effectively, leading to increased adsorption capacity and removal efficiency. The reported removal efficiencies were calculated from the residual PHY concentration measured in the final collected effluent after the complete influent solution volume had passed through the column.

#### 3.3.3 Effect of the solution temperature

**Fig. 14:**
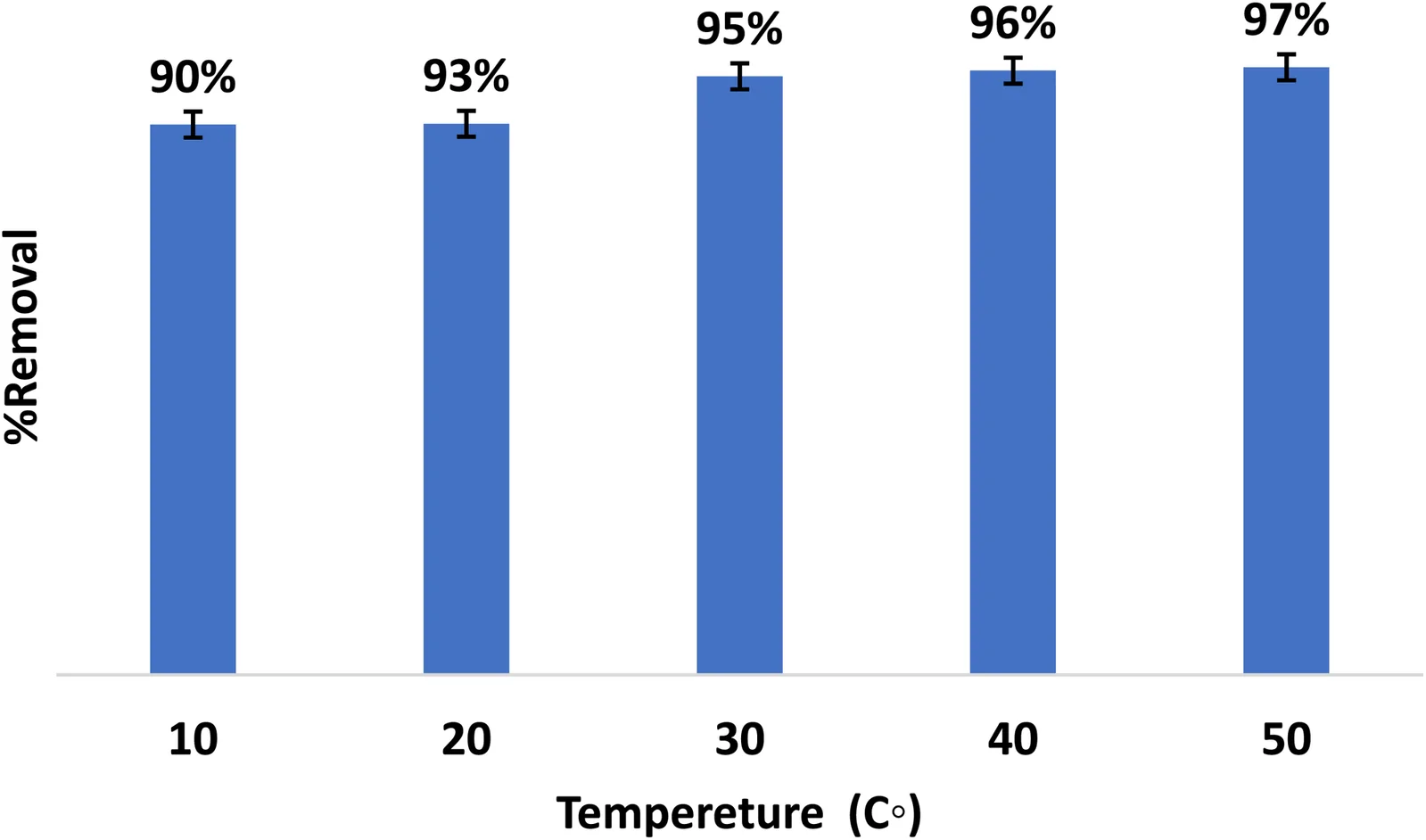
Effect of temperature on the removal efficiency of PHY in a continuous-flow system. Experimental conditions: initial influent concentration = 50 mg L^−1^, adsorbent mass = 6 g, bed height = 8 cm, flow rate = 10 mL min^−1^, regeneration temperature = 550 °C.

The effect of the solution temperature on column adsorption was evaluated over a range of 10–50 °C, while the other operating conditions remained the same (initial concentration of 50 ppm, flow rate of 10 mL min^−1^, adsorbent mass of 6 g, column height of 8 cm, and solution volume of 100 mL) (Fig. 14). As shown in Table 5, the removal efficiency increased slightly with increasing temperature, from 90% at 10 °C to approximately 97% at 50 °C. Such a trend shows that adsorption is an endothermic process, which is in line with the thermodynamic results obtained from the batch experiments reported earlier. At higher temperatures, the movement of PHY molecules is likely facilitated, as is their diffusion to the adsorption sites inside the porous structure of the Mnt/sand composite.

#### 3.3.4 Effect of column height (bed height)

The effect of bed height on the adsorption performance was investigated under continuous-flow conditions. To evaluate the influence of bed height independent of adsorbent loading, the adsorbent mass was kept constant at 3 g, while different bed heights (4 and 8 cm) were achieved using columns with different internal diameters.

Column height (bed height) significantly influenced the adsorption efficiency of the continuous-flow system. As presented in Table 5, higher removal efficiency and greater adsorption capacity were observed for the columns with greater bed heights. Such an increase in efficiency is due to an increased amount of time for the influent solution to spend in the column, resulting in better contact between the molecules of PHY and the active sites of the Mnt/sand composite. In addition, a taller bed creates a larger mass transfer zone, ensuring more efficient use of the adsorbent until breakthrough occurs.

**Fig. 15:**
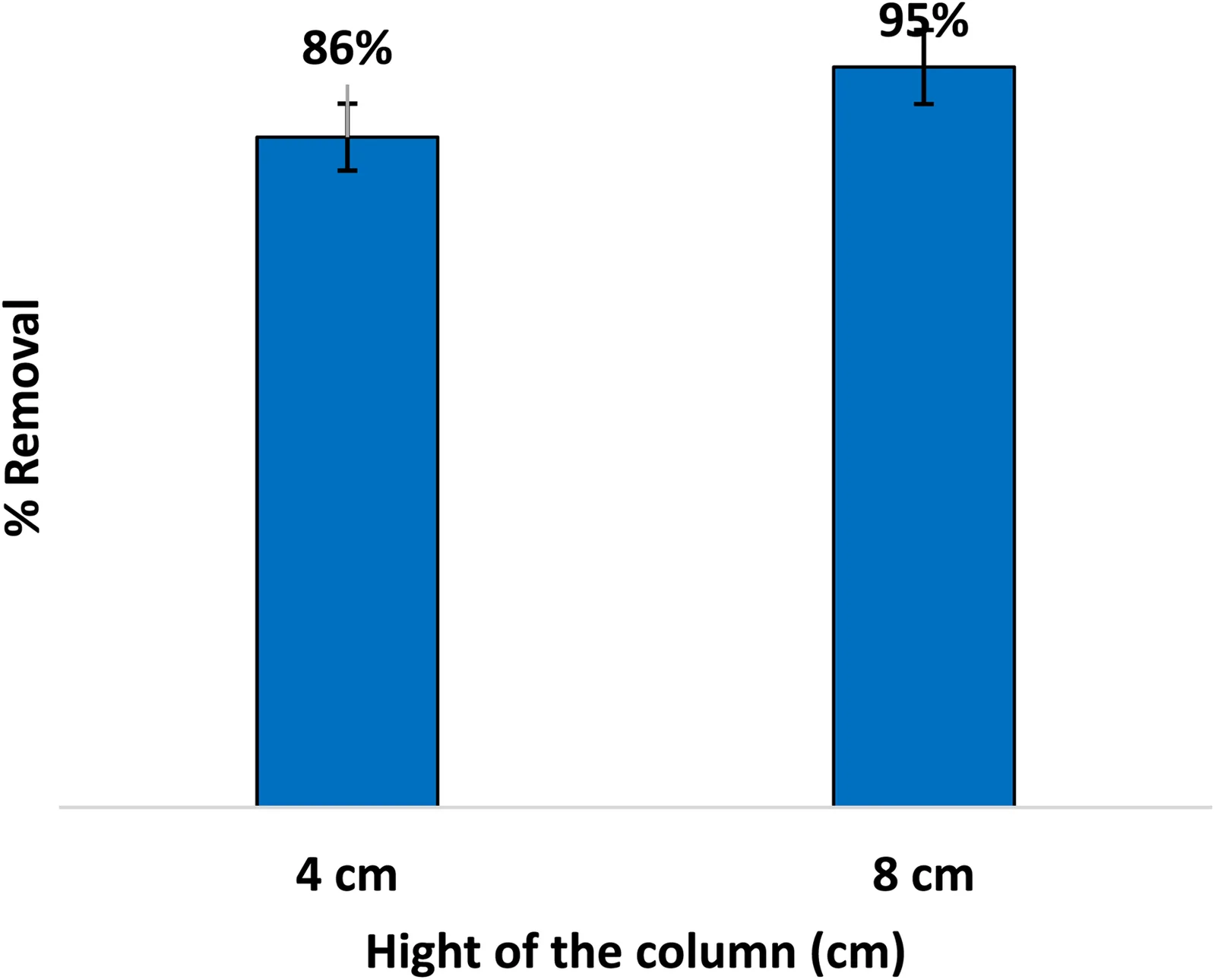
Effects of bed height (4 and 8 cm) on the removal efficiency of PHY in a continuous-flow system when the adsorbent mass was constant at 3 g. Experimental conditions: *C*_0_ = 50 mg L^−1^, flow rate = 4 mL min^−1^, and regeneration temperature = 550 °C.

Thus, the differences in the efficiency of the adsorption process were due mainly to changes in the bed height and hydrodynamics of the fluid flow and not to different adsorbent loadings (see Fig. 15). In general, an increase in the bed height with a constant adsorbent mass positively affected the efficiency of the column.

#### 3.3.5 Breakthrough curve analysis

**Fig. 16:**
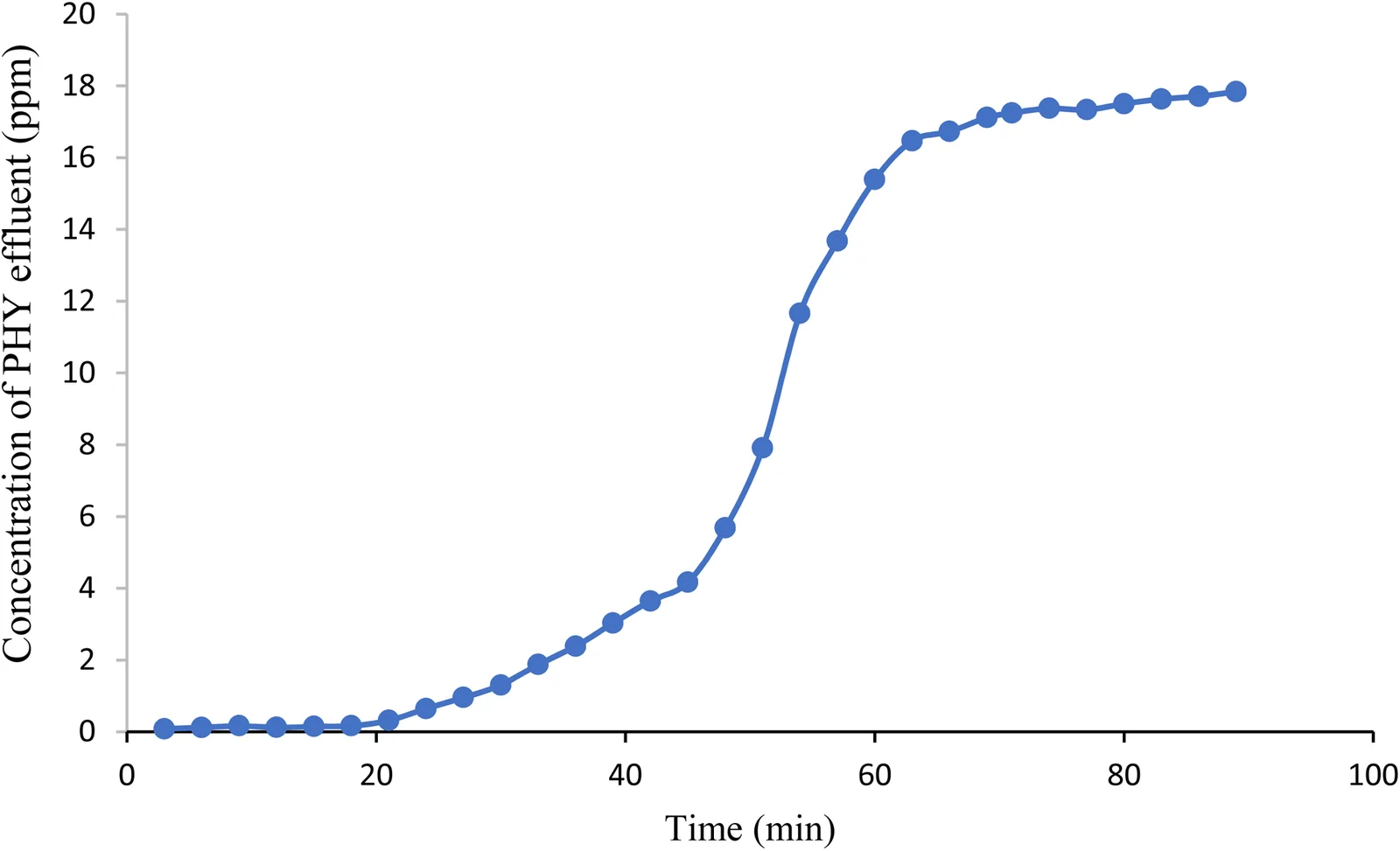
Breakthrough curve of PHY removal by the Mnt/sand filtration column.

A representative breakthrough test was performed at a nominal influent concentration of 20 mg L^−1^ for PHY, a solution volume of 200 mL, a flow rate of 2 mL min^−1^ and a weight of 3.0 g of Mnt/sand (Fig. 16). The breakthrough graph shows that the effluent concentration increased gradually as the packed bed approached saturation. This trend is common in fixed-bed adsorption processes since the mass transfer zone moves gradually along the bed as the active sites become saturated.

From the numerical analysis of the breakthrough profile, the breakthrough time at *C*_*t*_/*C*_0_ = 0.10 was 33.75 min, corresponding to a treated volume of 67.50 mL. The adsorption capacity at breakthrough was 0.439 mg g^−1^, while the cumulative capacity at the final measurement time of 89 min was 0.723 mg g^−1^. Because the recorded curve reached *C*_*t*_/*C*_0_ = 0.8919 but did not directly reach the 0.95 exhaustion criterion, no directly observed experimental exhaustion time or exhaustion capacity was assigned.

The Thomas model was applied as a representative fixed-bed model in the (Fig. S5.1–S5.3). The model gave *k*_Th_ = 5.14 × 10^−3^ L mg^−1^ min^−1^, *q*_Th_ = 0.758 mg g^−1^, and *R*^2^ = 0.9626. The Thomas capacity was therefore treated as a model-derived saturation/exhaustion capacity, yielding a bed utilization efficiency of 57.9% on the basis of *q*_b_/*q*_Th_. The normalized Thomas curve also showed acceptable agreement with the experimental breakthrough profile (*R*^2^ = 0.961; RMSE = 0.0736), supporting the use of the model as a preliminary description of the dynamic adsorption behavior. The complete dataset, calculation tables, and model plot are provided in SI Section S5.

#### 3.3.6 Regeneration and reusability in column systems

Thermal regeneration was conducted to examine the reusability of the PHY-loaded Mnt/sand composite when it was used under a continuous flow regime. The thermal decomposition profile of an organic molecule absorbed in the matrix could differ from that of a pure molecule because of changes in the surrounding environment, limited movement of the molecules, and effects on the bonding strength caused by surface interactions. Thus, while thermogravimetric analysis of pure PHY revealed complete decomposition at approximately 650–700 °C, this temperature is not the minimum needed to regenerate the PHY-loaded Mnt/sand.

To determine the right temperature for regeneration, the spent Mnt/sand composite was thermally treated at temperatures between 350 and 650 °C. The PHY removal efficiencies after regeneration at 350, 450, 500, 550, 600, and 650 °C were 97.37%, 98.12%, 98.19%, 98.24%, 98.25%, and 98.24%, respectively. It is clear from the low variability of these parameters that increasing the regeneration temperature above 550 °C does not result in any significant advantages in terms of the adsorption efficiency. Thus, 550 °C can be used as an effective temperature for the regeneration process, which allows high adsorption efficiency to be obtained without extra thermal treatments. This choice of temperature was additionally confirmed by the results of FT-IR analysis of the regenerated sample, in which the bands associated with PHY molecules were not detected after thermal treatment at 550 °C.

While thermal regeneration at 550 °C for 2 hours proved to be an effective method under the current study conditions, it is still a rather energetically costly operation. The practical sustainability of this regeneration approach depends on several scale-dependent factors, including the reactor configuration, adsorbent loading, heat-transfer efficiency, insulation, heating rate, heat recovery, and energy source used. Accordingly, the adsorption–thermolysis process should be considered in terms of the balance between regeneration energy demand, extended adsorbent lifetime, avoidance of chemical regenerants, and reduction in secondary waste generation. Further optimization of the regeneration temperature, treatment time, and heating strategy is important for improving the practical feasibility of this process.

A major operational advantage of the fixed-bed configuration is that the adsorbent can be regenerated and reused without an additional solid–liquid separation step. After each adsorption run, the packed column containing the PHY-loaded Mnt/sand composite was thermally regenerated at 550 °C for 2 h. Reusability experiments were conducted using 6 g of Mnt/sand packed in an 8 cm column, while 200 mL of 50 mg L^−1^ PHY solution was passed through the column at a flow rate of 10 mL min^−1^.

The regenerated column maintained stable adsorption performance over repeated adsorption–regeneration cycles. The PHY removal efficiency was 90% in the first cycle, followed by 89%, 88%, 89%, 87%, and 89% in the subsequent cycles. Overall, the removal efficiency remained within a narrow range of 87–90%, indicating that the Mnt/sand composite retained most of its adsorption performance after repeated thermal treatment. These results demonstrate the thermal stability and reusability of the composite and support its potential application in repeated fixed-bed water-treatment operations.

**Fig. 17:**
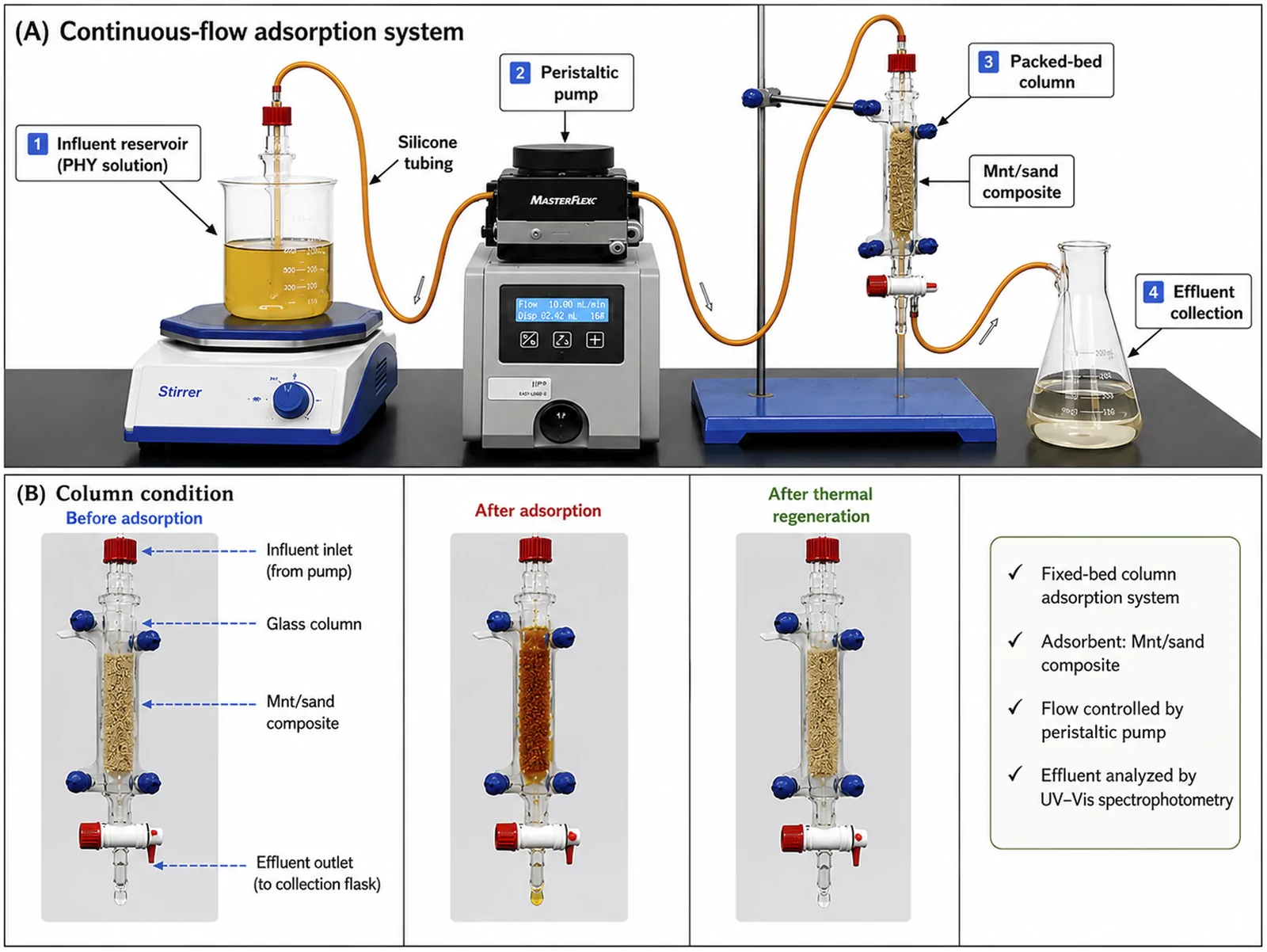
(A) Continuous-flow fixed-bed adsorption system used for phenazopyridine removal using the Mnt/sand composite. (B) Column conditions before adsorption, after adsorption, and after thermal regeneration.

The visual transformations that occurred during the adsorption and thermal regeneration cycles are presented in Fig. 17. The initial Mnt/sand sample appeared to be light in color prior to adsorption. Upon exposure to the PHY solution, the packed sample acquired an orange-brown color, indicating the presence of PHY in the bed. Thermal regeneration at 550 °C resulted in partial return of the original color of the adsorbent, which confirmed that the adsorbed phytochemicals were removed.

Generally, the regeneration of the column indicates that the Mnt/sand composite can be regenerated thermally without decreasing the efficiency of PHY removal. More research should be conducted regarding long-term breakthrough tests, exhaustion curves, repeated runs of the column, and optimization of regeneration.

### 3.4 Molecular-level mechanism of PHY adsorption and thermally assisted decomposition on Mnt/sand

The molecular simulation technique and computational settings are detailed in Section 2.7. DFT calculations along with GFN*n*-xTB preoptimization were carried out to study the interaction between PHY and the montmorillonite surface and to gain insight into structural modifications related to the adsorption and thermal regeneration processes. Preliminary structure optimization was performed by the GFN*n*-xTB semiempirical method, which was subsequently refined by the DFT method employing the DMol^3^ module with the PBE functional and DNP basis set.^47–50^

There were also some close interactions between PHY and Mnt, which geometrically agree with electrostatic attraction and hydrogen bonding. The approach of the amino and azo groups toward oxygenated regions of the Mnt surface indicates the possibility of multibond formation during the course of adsorption through the formation of a number of noncovalent contacts. To evaluate the strength of the interaction, the adsorption energy was computed as follows: *E*_ads_ = *E*_Mnt/PHY_ − (*E*_Mnt_ + *E*_PHY_). The corresponding energies of Mnt, isolated PHY, and the adsorption complex PHY/Mnt were calculated to be −552.022958, −43.86170907, and −596.0119773 hartree, respectively, leading to *E*_ads_ = −0.127310187 hartree or, in kcal mol^−1^ or electronvolts (as reported in subliminally S6), −334.25 kJ mol^−1^ or −3.46 eV. This proves the energetically favorable nature of the adsorption process for PHY molecules on the Mnt surface according to the chosen DFT model. It should be noted, however, that this stabilization cannot be attributed exclusively to electrostatic attraction but rather should be considered a consequence of electrostatic attraction, hydrogen bonding, van der Waals interactions, surface-induced polarization, and other noncovalent interactions in the adsorption complex.

Although the calculated negative adsorption energy confirms that PHY adsorption on the Mnt surface is energetically favorable, the value should be interpreted with caution because dispersion correction was not included in the present GGA-PBE calculations. Therefore, the DFT results qualitatively support the proposed adsorption mechanism, but dispersion-corrected calculations are needed in future work to quantify the contribution of long-range van der Waals interactions more accurately.

The structure of the optimal adsorption conformation implies that the stabilization of the PHY–Mnt complex is based on hydrogen bonding. Specifically, hydrogen bonds were formed between the hydrogens of the amino (–NH_2_) groups of the PHY molecule and oxygen atoms connected to the siloxane (Si–O) structure of the montmorillonite surface. Furthermore, other interactions were noted to occur between the azo group (–N=N–) and the oxygen atoms at the surface. These interactions ensure stabilization of the adsorbed complex but cause redistribution of the electron density within the PHY molecular structure. This could impact the polarity of bonds and the stability of molecules, especially those containing nitrogen.

Thus, the findings from the DFT analysis directly confirm the presence of hydrogen bonds as stabilizing forces, and the FT-IR data serve as experimental evidence for the involvement of PHY functional groups in the adsorption process. Taken together, this information confirms the occurrence of a multiinteraction adsorption mechanism rather than a simple electrostatically driven adsorption process.

**Fig. 18:**
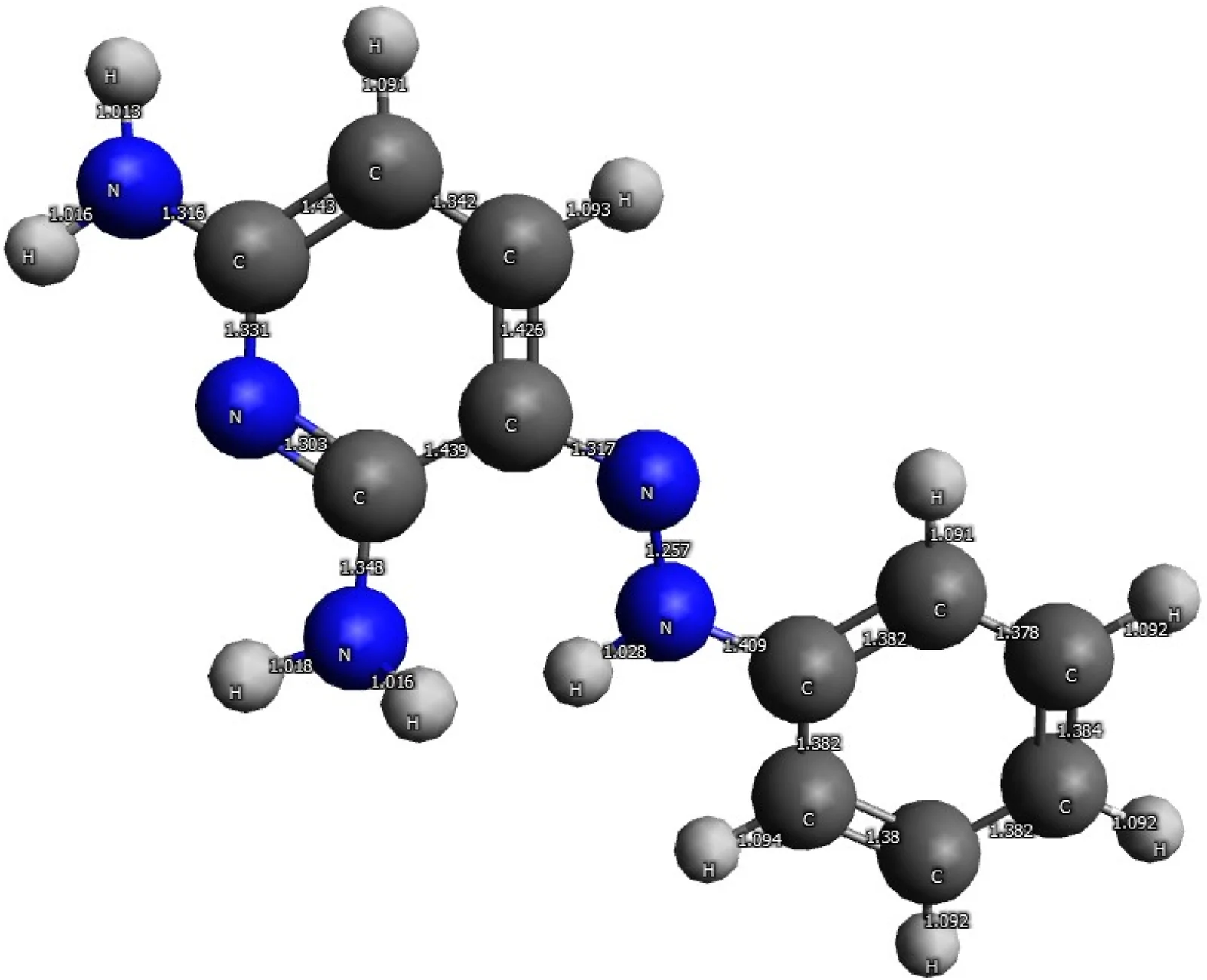
Phenazopyridine structure in the free state (planar structure), showing selected bond lengths of the phenazopyridine molecule before adsorption.

A comparison of the geometrical structures of free and adsorbed PHY molecules revealed significant structural changes during adsorption. Specifically, the free PHY molecule had a highly planar molecular structure because of aromatic conjugation (Fig. 17), whereas the adsorbed one was characterized by significant geometrical distortions because of anchoring interactions on the surface of the montmorillonite (Fig. 18). Such geometrical deformations can be considered partial destruction of the π-electron delocalization and internal electronic distribution of the molecule.

To further investigate these structural changes, bond length analysis was performed for selected bonds before and after adsorption (Table 6). The results revealed moderate elongation for several carbon–carbon and nitrogen-containing bonds after adsorption. For example, the N1–N2 bond length increased from 1.257 to 1.276 Å, whereas the N5–C7 bond length increased from 1.316 to 1.337 Å. Similar but smaller elongations were also observed for aromatic carbon–carbon bonds. These bond length variations suggest the occurrence of adsorption-induced structural perturbation and possible weakening tendencies within the PHY molecule following interaction with the montmorillonite surface.

**Table 6:** Selected PHY bond lengths before and after adsorption according to GFN*n*-xTB/DFT-supported molecular simulations

Bond symbol	Bond length before adsorption (Å)	Bond length after adsorption (Å)	Degree of elongation (Å)
C1–C2	1.382	1.395	0.013
C1–C3	1.382	1.395	0.013
C8–C10	1.342	1.355	0.013
C9–C11	1.439	1.453	0.014
N3–C9	1.303	1.319	0.016
N1–N2	1.257	1.276	0.019
N5–C7	1.316	1.337	0.021

The observed structural changes were more pronounced for nitrogen-containing bonds, which are generally more sensitive to local electronic perturbations. These findings suggest the possible activation of specific bonds rather than definitive bond cleavage. Furthermore, the combined effects of hydrogen bonding, electrostatic interactions, and surface-induced polarization may contribute to reducing the stability of the adsorbed PHY molecule during subsequent thermal treatment.

From a mechanistic perspective, the adsorption process may therefore contribute to molecular preactivation, whereby adsorption-induced structural and electronic perturbations facilitate subsequent thermally assisted degradation. In this context, compared with free molecules, the Mnt surface may have increased the susceptibility of PHY molecules to thermolysis at lower temperatures.

These computational observations are consistent with the experimental findings, which demonstrated effective regeneration of the Mnt/sand composite at relatively moderate regeneration temperatures (∼550 °C). From the point of view of practice, the capability of efficient regeneration at diminished thermal parameters can be considered significant, as it can lead to decreased energy costs and enhanced operational sustainability of the adsorption–thermolysis cycle.

**Fig. 19:**
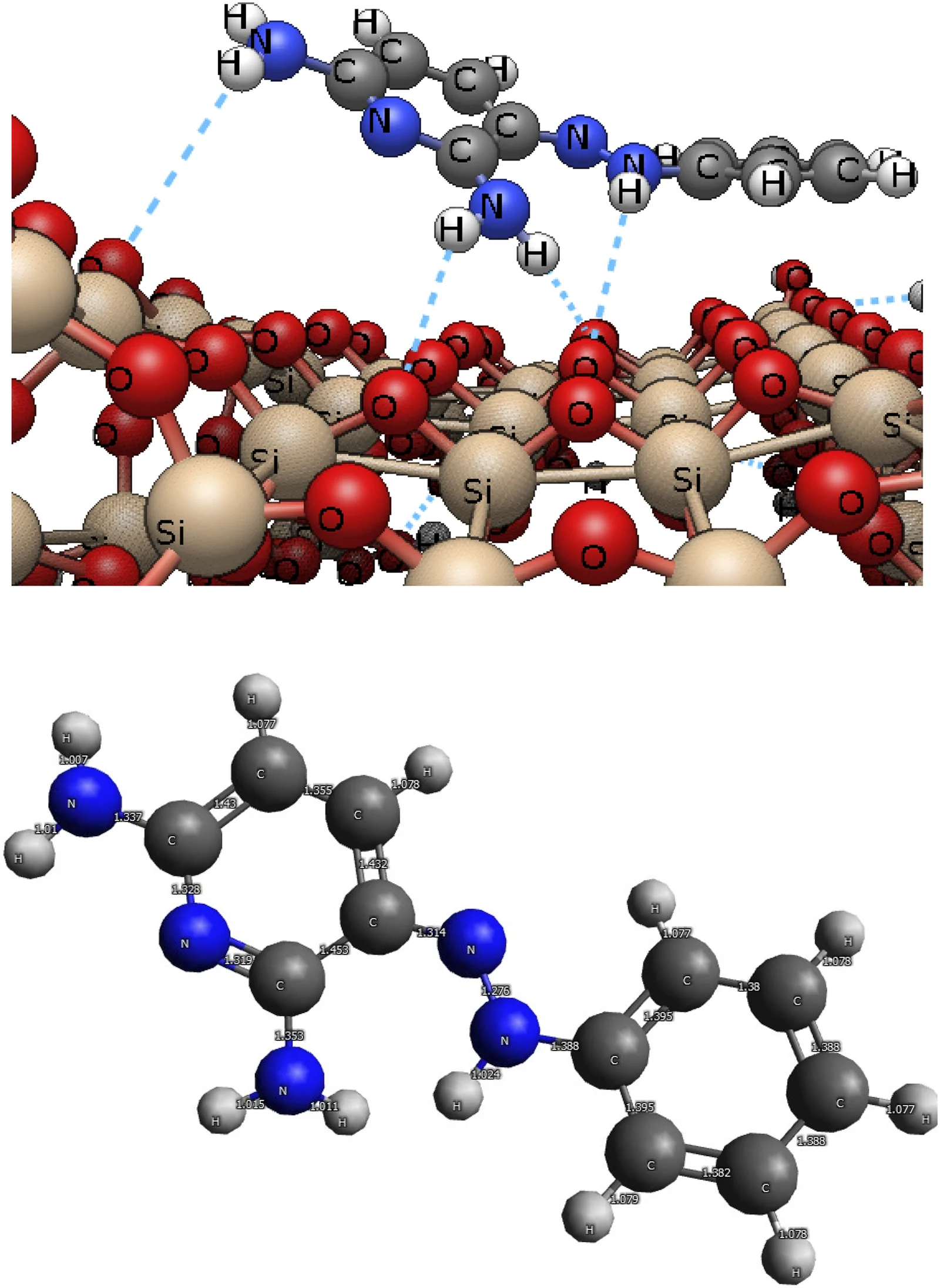
Hydrogen bonds formed between the phenazopyridine molecule and the Mnt surface. The adsorbed PHY structure is distorted relative to that of the free molecule and shows selected bond lengths after adsorption.

On the basis of the results of the analysis of theoretical and experimental data, one can conclude that the montmorillonite surface is active in the regulation of the adsorption properties and structural stability of PHY molecules because of several types of intermolecular interactions. Structural distortions, which have been obtained from DFT calculations, can favor the processes of thermally induced decomposition (Fig. 19).

**Fig. 20:**
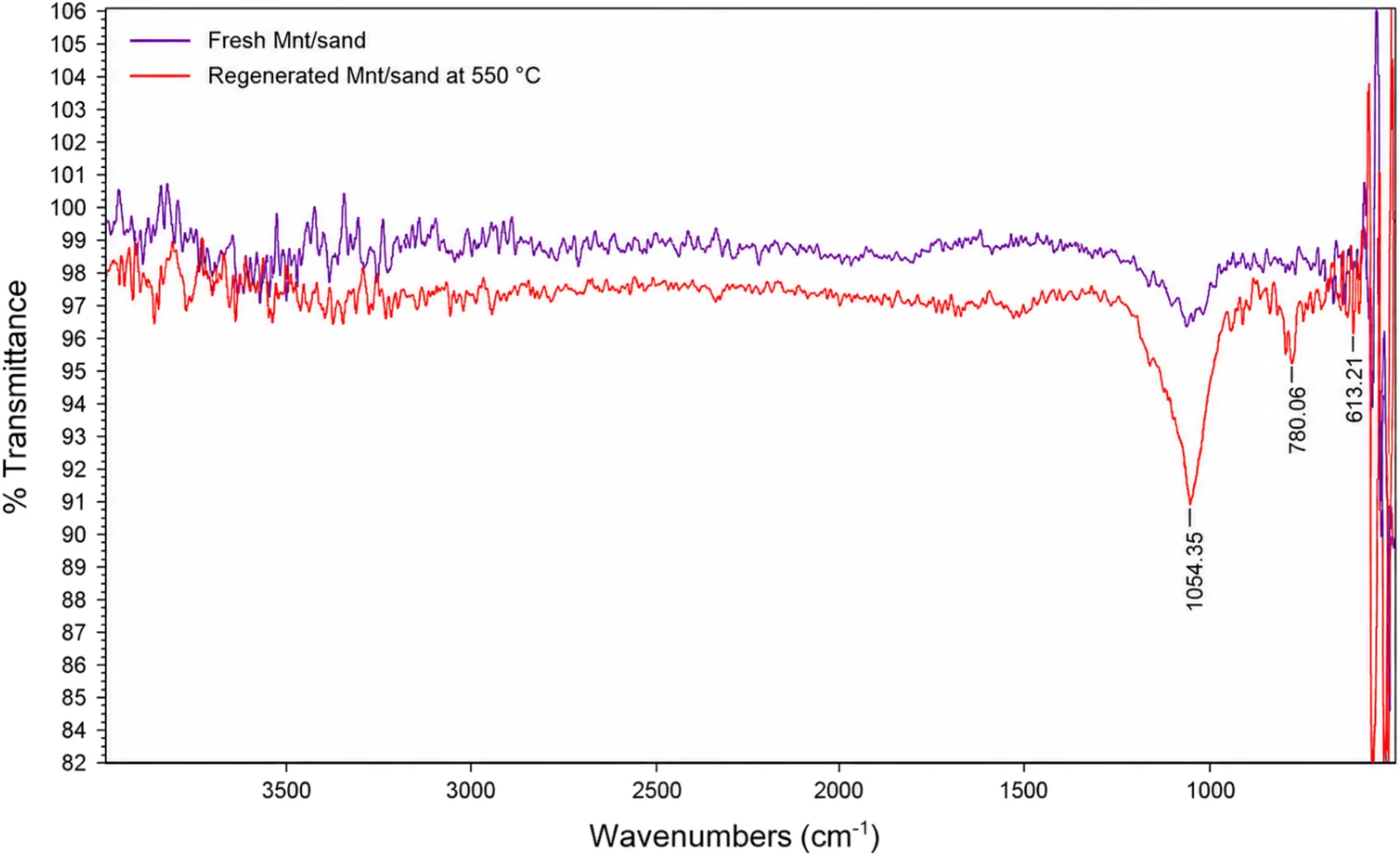
FT-IR spectra of regenerated Mnt/sand at 550 °C and fresh Mnt/sand samples.

FT-IR analysis of the regenerated adsorbent provides additional evidence for the effectiveness of the selected regeneration temperature. After thermal treatment at 550 °C, the main PHY-related absorption bands disappeared, whereas the characteristic bands of the Mnt/sand composite were retained (Fig. 20). This indicates effective decomposition or removal of adsorbed PHY residues from the composite surface at 550 °C, even though complete decomposition of free PHY in TGA occurs close to 650 °C. The difference between free and adsorbed PHY behavior is consistent with the proposed surface-assisted activation, in which hydrogen bonding, electrostatic interactions, and other secondary interactions with Mnt perturb selected PHY bonds, as indicated by the changes in the length of the DFT bond. Thus, the selection of 550 °C is supported by regeneration-temperature screening, FT-IR disappearance of PHY-related bands, stable adsorption performance after repeated cycles, and computationally suggested bond weakening. Nevertheless, FT-IR confirms the disappearance of adsorbed organic residues from the regenerated adsorbent surface but does not quantify mineralization products such as CO_2_, H_2_O, or NO_*x*_ alone.

### 3.5 Practical applicability, matrix effects, and study limitations

However, even though the efficiency of PHY adsorption from artificial water solutions under laboratory conditions was shown in the present study, the efficiency of the Mnt/sand composite could differ when it is used to adsorb PHY from real wastewater samples. First, real wastewater samples usually contain different ions, natural organic material, suspended particles, and other organic compounds that might influence the process of adsorption. Other cations or anions can interact with the active centers of the Mnt surface, change its electrical double layer or the surface charge of the composite, or affect electrostatic interactions between the adsorbent and adsorbate. Organic materials, such as humic and fulvic acids, may also compete for adsorption sites with PHY.

The coexistence of pharmaceuticals, dyes, and various organic micropollutants could also cause competition for adsorption sites through electrostatic attractions, hydrogen bonding, π-interactions, van der Waals attractions, and diffusion to accessible pores and interlayers. On the other hand, some dissolved ions may decrease electrostatic repulsion or cause an ion bridging effect and facilitate adsorption depending on certain conditions. Thus, the current results should be considered as proof-of-concept adsorption and desorption studies conducted under laboratory conditions only. The validation of the results with wastewater is necessary prior to practical application. In future research, the influence of ionic strength, various interfering ions, natural organic matter, suspended particles, and coexisting pharmaceutical pollutants should be investigated using actual wastewater and artificial multicomponent solutions.

Although BET specific surface area measurements were performed and confirmed the contribution of Mnt to the surface area of the Mnt/sand composite, additional surface and textural analyses are still needed for a more complete description of the adsorbent. Raman spectroscopy and X-ray photoelectron spectroscopy (XPS) were not performed in the present study. Raman spectroscopy could provide further information on surface bonding and adsorbate–adsorbent interactions, while XPS could clarify elemental composition, chemical states, and binding-energy shifts before and after adsorption/regeneration. In addition, full N_2_ adsorption–desorption analysis would provide more detailed information on pore volume and pore-size distribution. Therefore, these complementary techniques are recommended for future work to further understand the surface chemistry, binding properties, and pore structure of the Mnt/sand composite.

Thermal regeneration was evaluated only at the laboratory scale using a conventional muffle furnace. Consequently, the present results should not be directly extrapolated to commercial-scale energy consumption, process economics, or environmental performance. Further work should investigate the feasibility of lower regeneration temperatures, shorter heating durations, optimized heating rates, heat recovery strategies, and alternative or renewable energy sources for thermolysis. The degradation products and extent of PHY mineralization should also be verified using complementary analytical techniques, such as evolved-gas analysis, total organic carbon analysis, gas chromatography–mass spectrometry, or thermogravimetric analysis coupled with mass spectrometry. Technoeconomic and life cycle assessments should be performed only after the regeneration conditions and process configuration have been validated at the pilot or larger scale.

The computational analysis was based on a representative PHY–Mnt adsorption configuration. However, despite the favorable adsorption energy, the current DFT study is based on the application of the GGA-PBE functional without a dispersion correction. Given that traditional PBE may underestimate long-range van der Waals forces, the current adsorption energy calculation should be considered as mechanistic evidence in terms of the computational model applied rather than a comprehensive description of adsorption thermodynamics. For future work, it would be appropriate to use dispersion-corrected approaches, namely, PBE-D or PBE-D3, and consider several orientations, protonation states, compositions, defects, and solvent effects.

The reproducibility of the preparation procedure was verified through the preparation of three independent Mnt/sand batches with yields of 95.0%, 93.0%, and 94.0%, resulting in an average yield of 94.0 ± 1.0% and an RSD of 1.06% (*n* = 3). Thus, this preparation procedure demonstrates very good reproducibility at the laboratory scale. However, scale-up could lead to increased variability because of other factors, such as the homogeneity of mixing, particle agglomeration, and heat exchange. Thus, further research needs to include larger preparation batches and evaluate the particle size distribution, mechanical stability and resistance to attrition, hydraulic properties, and reproducibility of the adsorption performance.

## 4. Conclusions

The current investigation provides evidence of the effectiveness of thermally regenerable montmorillonite/sand (Mnt/sand) composites in removing phenazopyridine (PHY) from aqueous solution. Batch adsorption tests revealed that the adsorption of PHY proceeded in accordance with pseudo-second-order kinetics and was well described using the Langmuir isotherm model, which implies monolayer adsorption on the active surface sites. Thermodynamic analysis revealed that the adsorption process was spontaneous and endothermic. The total-mass-based Langmuir capacity includes the mass of both the active Mnt phase and the sand substrate; thus, the practical significance of the Mnt/sand composite cannot be evaluated solely by the gravimetric capacity.

In turn, continuous flow tests demonstrated the possibility of using Mnt/sand composite in fixed-bed mode and proved that the performance of the column depends on the flow rate and bed height. In the case of the representative breakthrough experiment, the breakthrough time was 33.75 min, the breakthrough capacity was 0.439 mg g^−1^, and the Thomas model accounted for a dynamic capacity of 0.758 mg g^−1^. The agreement between the experimental data and theoretical calculations was high (*R*^2^ = 0.963). The respective bed utilization efficiency was 57.9%. As the experimentally obtained breakthrough curve did not reach the predetermined exhaustion limit of *C*_*t*_/*C*_0_ = 0.95 but stopped at *C*_*t*_/*C*_0_ = 0.8919, the value of capacity obtained using the Thomas model was considered the saturation capacity rather than the exhaustion capacity. These findings support the feasibility of using the composite in continuous treatment systems, but longer breakthrough experiments and validation under wider operating conditions are needed for scaling up.

Thermal regeneration experiments revealed that the Mnt/sand composite can be recycled after heating to 550 °C within 2 h while preserving the high stability of the PHY removal efficiency during successive adsorption/regeneration cycles. The choice of 550 °C was substantiated by the results of screening experiments on the influence of regeneration temperature on adsorption, FT-IR analysis revealing the disappearance of the characteristic bands associated with PHY after regeneration, and DFT calculations predicting preferable PHY–Mnt interactions and adsorption-driven structural modifications. Nevertheless, thermal regeneration is a costly process, and the sustainability of the adsorption/thermolysis process must be estimated considering the energy costs of regeneration, lifetime extension of adsorbents, absence of chemical reagents for regeneration and generation of less secondary waste. Therefore, further improvement of the parameters of thermal regeneration is necessary.

In general, the Mnt/sand composite represents a promising tool for the integration of adsorption, regeneration, and fixed-bed operations for the treatment of pharmaceutical-containing water. Nevertheless, the present study was conducted using synthetic PHY solutions; therefore, future investigations should evaluate the composite in real wastewater and multicomponent systems to assess the effects of competing ions, natural organic matter, suspended solids, and coexisting pharmaceutical contaminants on the adsorption performance and regeneration efficiency.

## Author contributions

The results presented in this work are derived from the thesis of Sh. M., which was conducted under the supervision of A. H. Z. and Sh. H. Z. Sh. H. Z. contributed to the conceptualization, visualization, and validation of the study. A. A. carried out part of the experimental work and assisted in data collection. S. Z., D. C., and T. K. performed the XRD, SEM, and EDS analyses. M. F. conducted the DFT computations. A. H. Z. & W. A. Z. were responsible for drafting and writing the manuscript. All the authors reviewed and approved the final version of the manuscript.

## Conflicts of interest

The authors declare that they have no known competing financial or nonfinancial interests that could have appeared to influence the work reported in this paper.

## Supplementary Material

RA-016-D6RA05599K-s001

## Data Availability

The data that support the findings of this study are available from the corresponding author upon reasonable request.
