# Supplementary material for: Thermally regenerable montmorillonite/sand composite for the adsorptive removal of phenazopyridine from aqueous solutions
Source: RSC Adv. 2026 Aug 5;16(39):44832–51. doi: 10.1039/d6ra05599k (PMC13439743; doi:10.1039/d6ra05599k)
Supplement: Supplementary file 1 [file RA-016-D6RA05599K-s001.pdf]

Supplementary Information (SI) for RSC Advances.
This journal is © The Royal Society of Chemistry 2026

Fig. S1: Adsorption Isotherms

Adsorption isotherm analysis was performed to evaluate the interaction between phenazopyridine (PHY) and the montmorillonite/sand (Mnt/sand) composite and to determine the adsorption capacity and surface properties of the adsorbent. Adsorption equilibrium data were obtained by measuring the equilibrium concentration of PHY in solution at different initial concentrations under constant experimental conditions (temperature 25–30 °C, pH 5, and adsorbent dose of 0.5 g per 100 mL). The equilibrium adsorption capacity (q_e) and equilibrium concentration (C_e) values were calculated and summarized in Table S1.

Table S1: *equilibrium concentrations and masses adsorbed for PHY onto Mnt/sand.*

C	C_e	q_e	1/C	$1/q_e$	Log C_e	Log q_e
10	0.98	1.98	1.01	0.50	0.01	0.29
20	2.35	3.69	0.42	0.27	0.37	0.56
30	4.81	5.62	0.20	0.17	0.68	0.75
40	8.80	6.18	0.11	0.16	0.94	0.79
50	16.12	7.05	0.06	0.14	1.20	0.84

Note: The q_e values in Table S1 are expressed on the basis of the total Mnt/sand composite mass. Because the composite contains 20 wt% active Mnt and 80 wt% sand support, the corresponding Mnt-normalized capacities can be obtained by dividing the total-mass-based values by 0.20. Accordingly, the Langmuir qmax of 8.98 mg g^{-1} based on the total composite mass corresponds to 44.90 mg g^{-1} when normalized to the active Mnt fraction. This normalized value is used only for comparison with clay-based adsorbents, whereas the total-composite capacity is retained for process-design and practical application discussion.

The experimental data were analyzed using the Langmuir and Freundlich adsorption isotherm models. The Langmuir model assumes monolayer adsorption on a homogeneous adsorbent surface with identical adsorption sites and no interaction between adsorbed molecules. The linearized form of the Langmuir equation is expressed as:

$$\frac{1}{q_e} = \frac{1}{K_L\, q_{max}} . \frac{1}{C_e} + \frac{1}{q_{max}} \qquad \text{……………………… (1)}$$

where (C_e) (mg.L^{-1}) is the equilibrium concentration of the adsorbate in solution, (q_e) (mg.g^{-1}) is the amount of adsorbate adsorbed per unit mass of adsorbent at equilibrium, (q_{max}) (mg g^{-1}) represents the maximum monolayer adsorption capacity, and (K_L) (L.mg^{-1}) is the Langmuir equilibrium constant related to the affinity between the adsorbent surface and the adsorbate molecules.

The Freundlich model describes adsorption on heterogeneous surfaces and multilayer adsorption behavior. The linearized Freundlich equation is given as:

$$logq_e = \log K_f + \frac{1}{n} logC_e \qquad \text{……………………… (2)}$$

where (K_F) represents the Freundlich adsorption capacity constant and (n) is an empirical parameter related to adsorption intensity.

The isotherm parameters were obtained from the slope and intercept of the corresponding linear plots, Fig. S1. The results indicated that the Langmuir model provided a better fit to the experimental data than the Freundlich model. The correlation coefficient for the Langmuir model was ($R^2 = 0.996$), which is closer to unity compared with that obtained for the Freundlich model. This result suggests that the adsorption of PHY onto the Mnt/sand composite occurs mainly through monolayer adsorption on a relatively homogeneous surface.

(a)

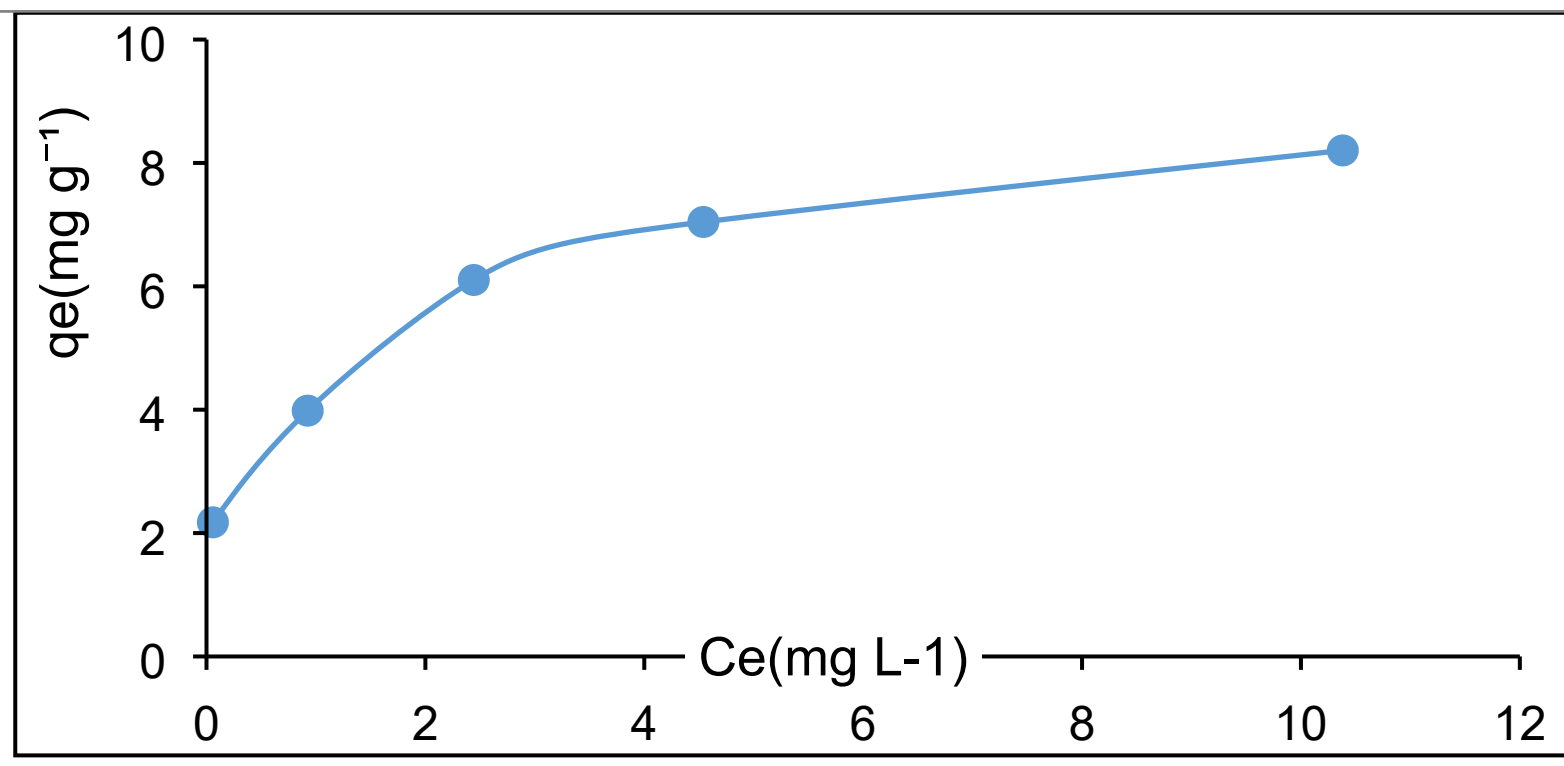

(b)

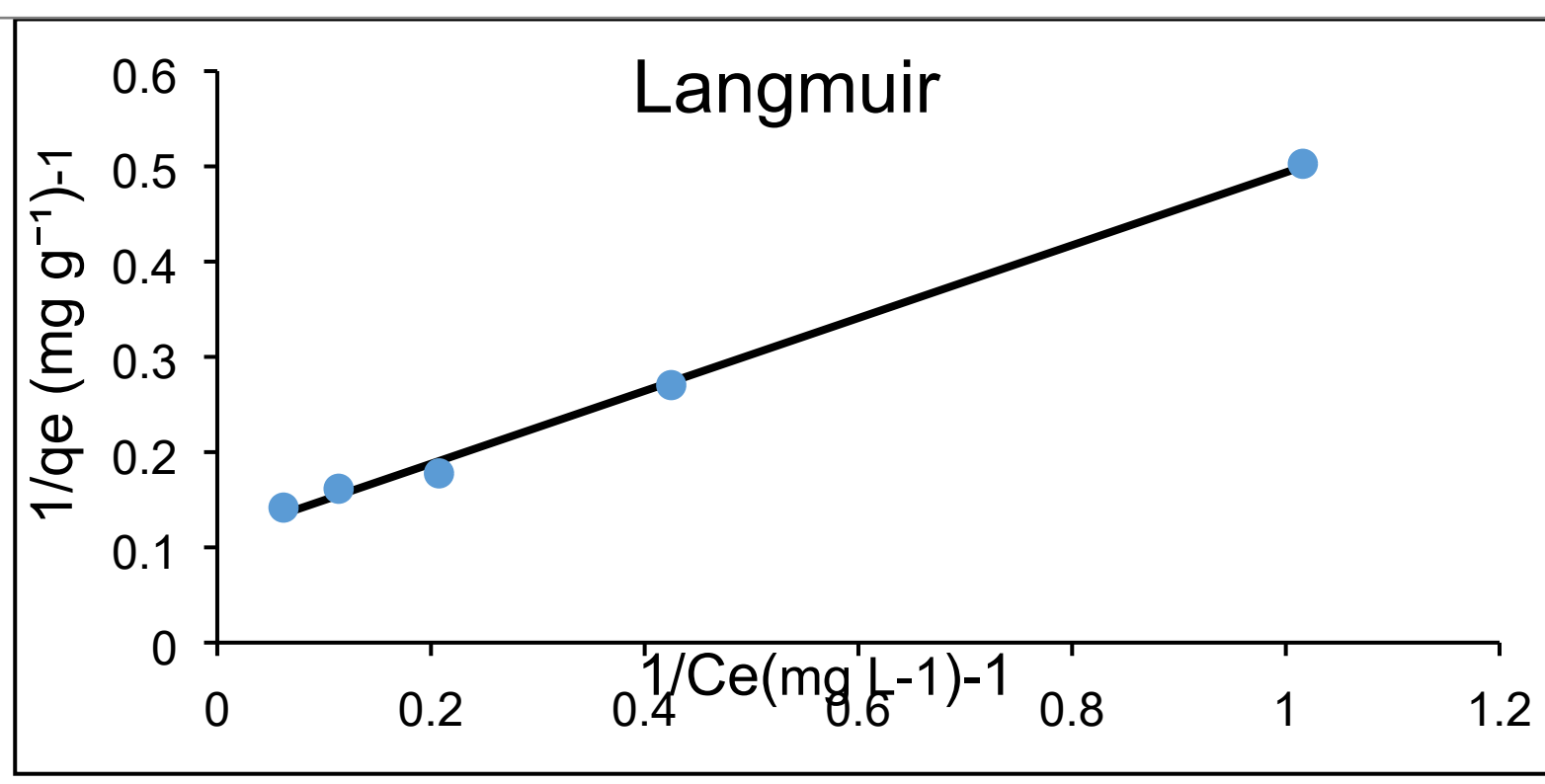

(c)

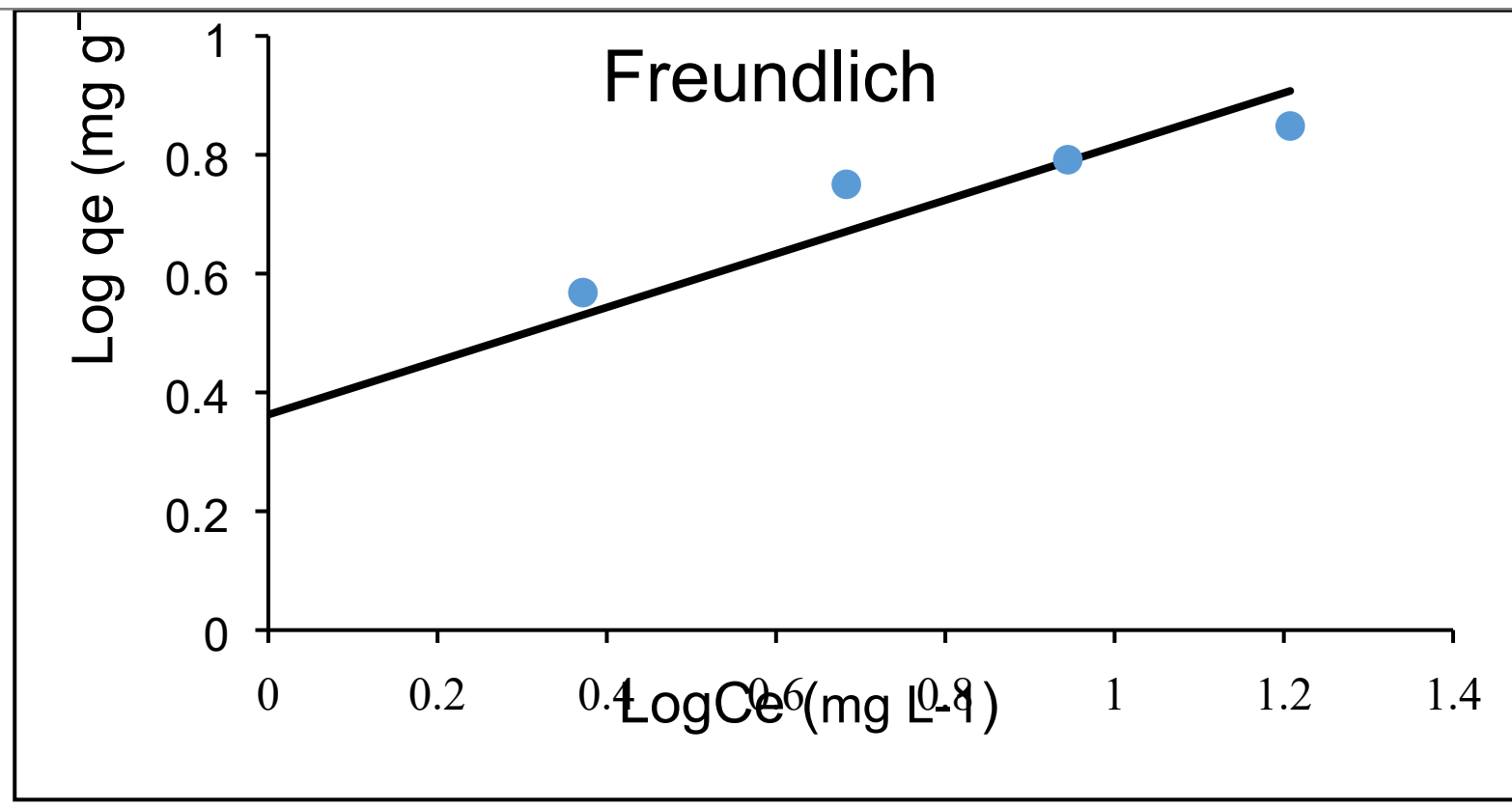

Fig. S1. (a) Equilibrium adsorption isotherm of PHY onto Mnt/sand (temperature: 25 °C, initial pH: 5, and solid/liquid ratio: 0.5 g/100 mL); (b) Langmuir isotherm; and (c) Freundlich isotherm.

Fig. S2: Adsorption Thermodynamics

Thermodynamic analysis was conducted to evaluate the feasibility and nature of the adsorption process. The thermodynamic parameters, including the standard Gibbs free energy change (ΔG°), enthalpy change (ΔH°), and entropy change (ΔS°), were determined from adsorption experiments performed at different temperatures ranging from 10 to 50 °C.

The distribution coefficient (K_d) was calculated according to the following relationship:

$$K_d = CAe/Ce \qquad \ldots\ldots\ldots\ldots\ldots\ldots (3)$$

where (C_{Ae}) represents the amount of adsorbate adsorbed on the solid at equilibrium and (C_e) is the equilibrium concentration of the adsorbate remaining in solution.

The Gibbs free energy change was determined using the equation:

$$\Delta G = -RT \ln K_d \qquad \ldots\ldots\ldots\ldots\ldots\ldots\ldots.. (4)$$

where (R) is the universal gas constant (8.314 J mol^{-1} K^{-1}) and (T) is the absolute temperature in Kelvin.

The values of enthalpy change (ΔH°) and entropy change (ΔS°) were obtained from the linear form of the Van't Hoff equation:

$$\text{Ln } K_d = -\Delta H°/RT + \Delta S°/R \quad \ldots\ldots\ldots\ldots\ldots\ldots\ldots (5)$$

A plot of (ln K_d) versus (1/T) was constructed, where the slope and intercept correspond to −ΔH°/R and ΔS°/R, respectively, Fig. S2. The calculated thermodynamic parameters are summarized in Table 2 of the main manuscript.

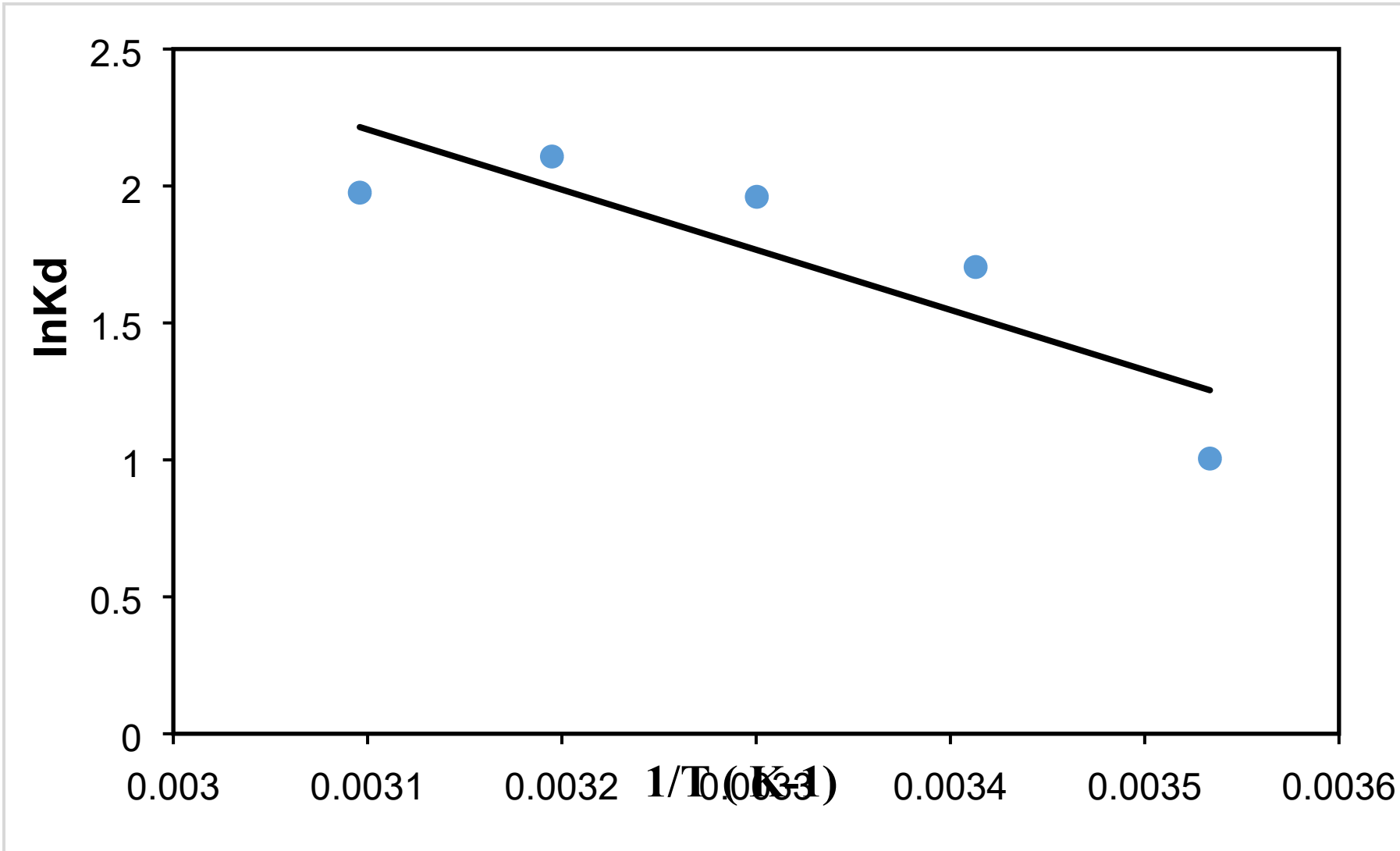

Fig. S2. Plot of ln Kd versus 1/T for ΔH° and ΔS° determination (30 mg L^{-1} PHY, 100 mL solution, 0.5 g Mnt/sand, pH 5).

Fig. S3: Adsorption Kinetics

Adsorption kinetics were investigated to better understand the rate and mechanism of PHY adsorption onto the Mnt/sand composite. Kinetic analysis provides valuable information regarding the adsorption mechanism and the rate-controlling steps involved in the adsorption process.

The experimental kinetic data were analyzed using three commonly applied models: the pseudo-first-order model, pseudo-second-order model, and intraparticle diffusion model.

The pseudo-first-order kinetic model, proposed by Lagergren, assumes that the rate of adsorption is proportional to the difference between the equilibrium adsorption capacity and the amount adsorbed at any time. The linearized form of this model is expressed as:

$$\log(q_e - q_t) = \log q_e - \frac{k_1}{2.303} t \qquad \text{……………………. (6)}$$

where (q_e) and (q_t) represent the adsorption capacities at equilibrium and at time (t), respectively, and (k_1) (min^{-1}) is the pseudo-first-order rate constant.

The pseudo-second-order model assumes that the adsorption rate is controlled by chemisorption involving electron sharing or exchange between the adsorbent and adsorbate. The linear form of this model is:

$$\frac{t}{q_t} = \frac{1}{k_2 q_e^2} + \frac{1}{q_e} t \qquad \text{……………….. (7)}$$

where (k_2) (g mg^{-1} min^{-1}) is the pseudo-second-order rate constant.

To examine the possibility of diffusion-controlled adsorption, the intraparticle diffusion model proposed by Weber and Morris was also applied:

$$q_t = k_i t^{1/2} + C \qquad \text{.......................... (8)}$$

where (k_i) is the intraparticle diffusion rate constant and (C) represents the boundary layer thickness, Fig. S3.

**(a)**	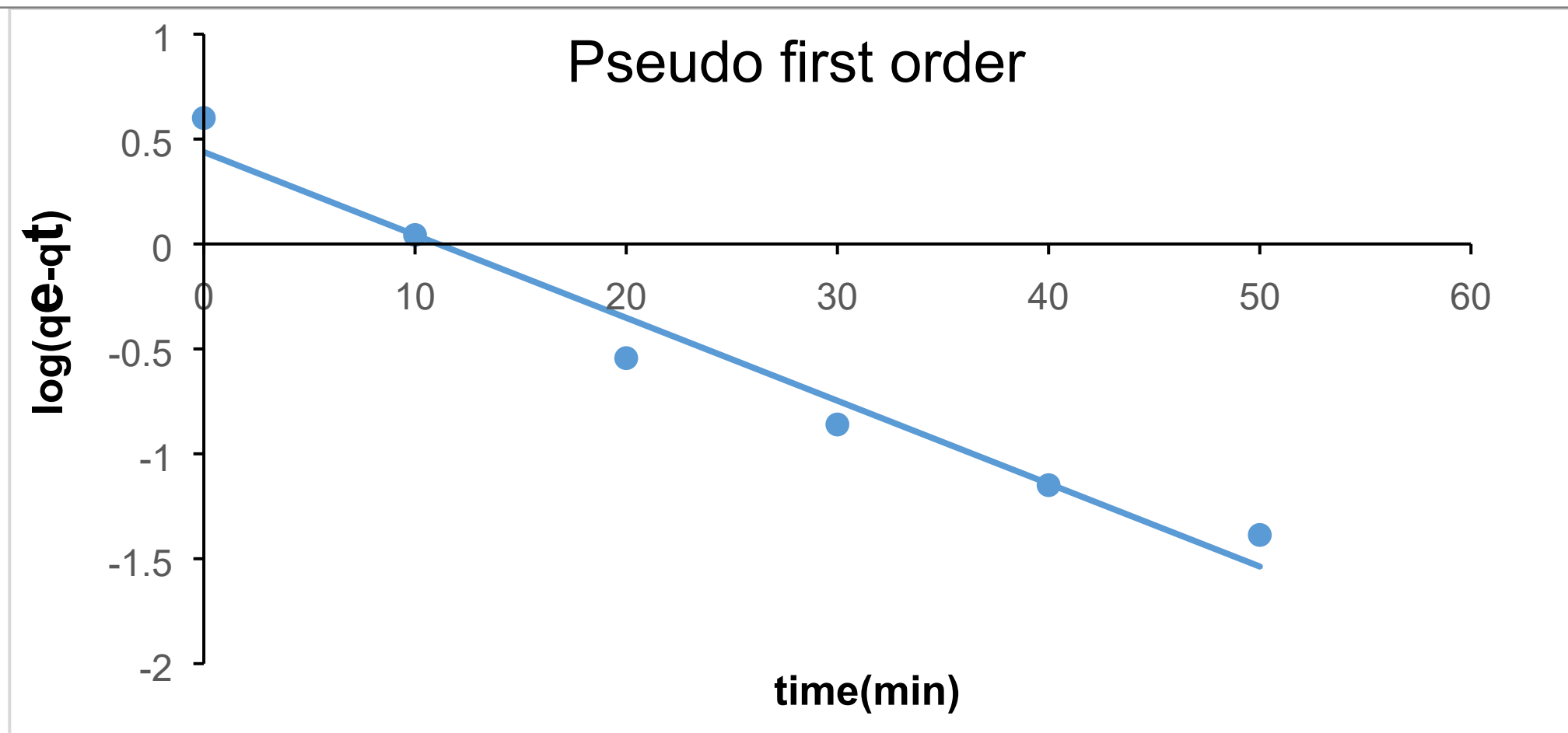
(b)	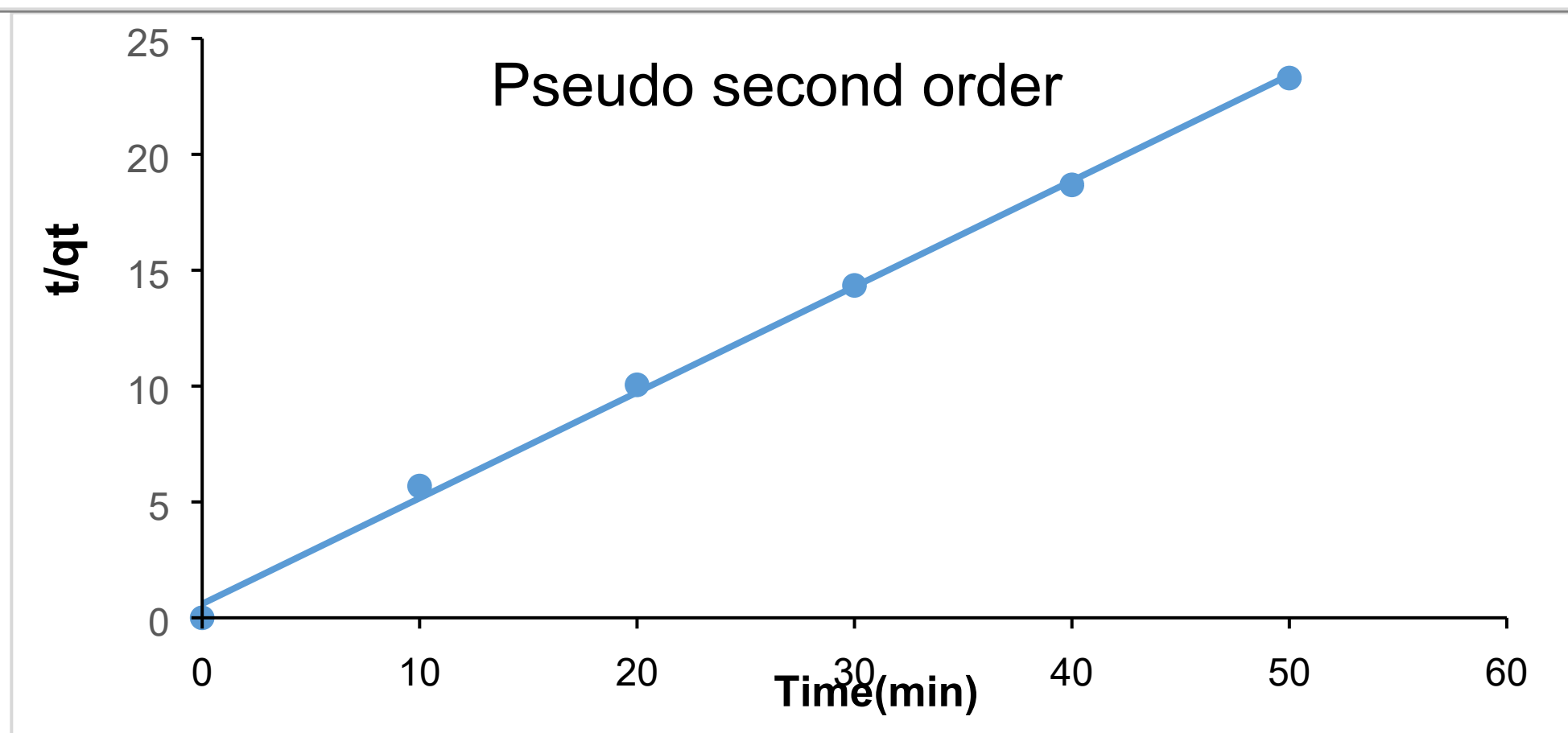

(c)

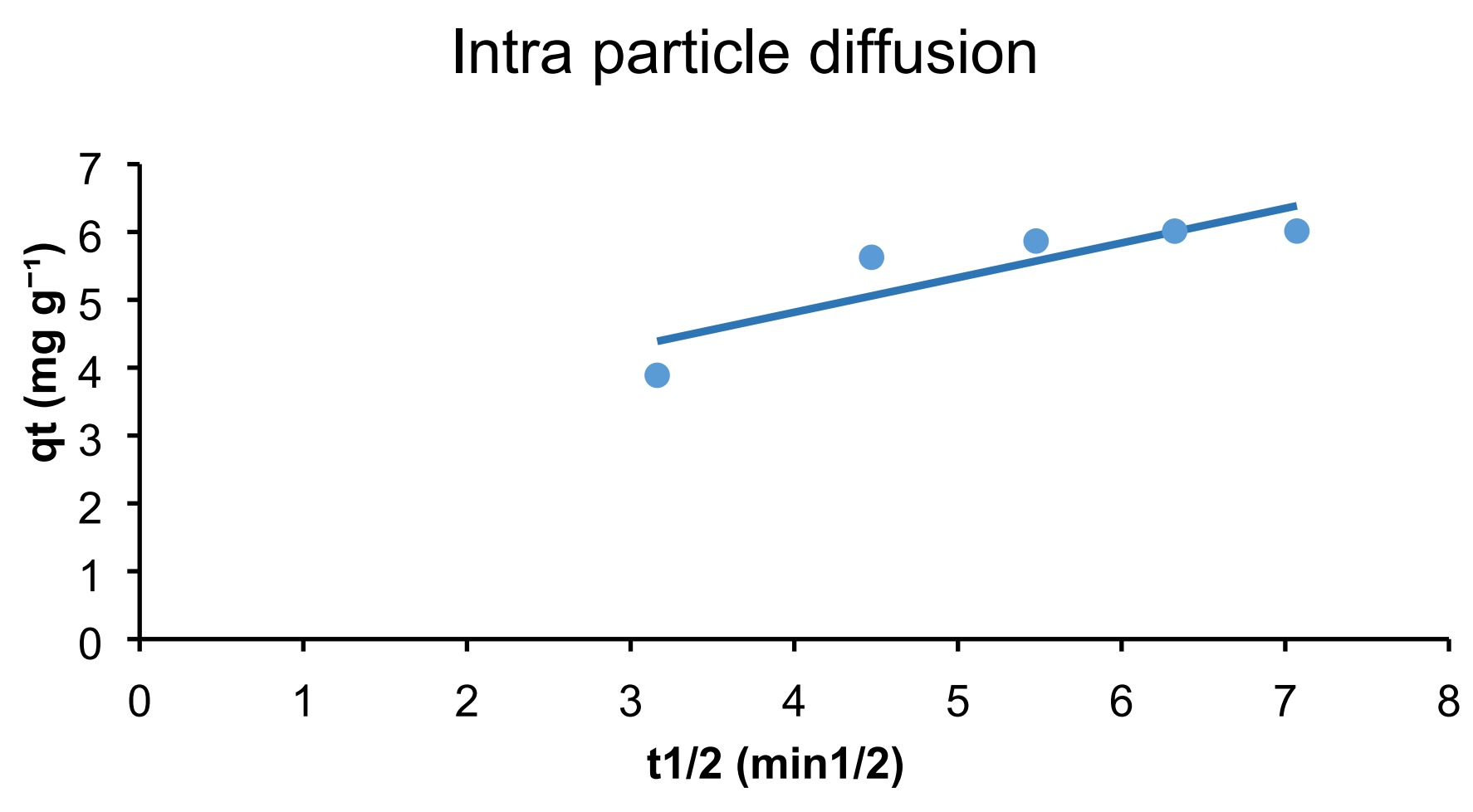

Fig. S3. Kinetic analysis of PHY adsorption onto Mnt/sand using (a) pseudo-first-order, (b) pseudo-second-order, and (c) Weber–Morris intraparticle diffusion models. The nonzero intercept and the regression line not passing through the origin indicate that intraparticle diffusion was not the sole rate-controlling step.

Fig. S4: Activation Energy Determination

The activation energy of the adsorption process was estimated using the Arrhenius equation based on the pseudo-second-order kinetic rate constants (k_2) obtained at different temperatures:

$$k = Ae^{-Ea/RT} \quad \text{.....................} \ (9)$$

which can be expressed in linear form as:

$$\ln k = \frac{-Ea}{RT} + lnA \quad \text{.......................} \ (10)$$

where k represents the pseudo-second-order rate constant (k_2), (A) is the pre-exponential factor, (E_a) is the activation energy, (R) is the universal gas constant, and (T) is the temperature in Kelvin. The activation energy calculated from the Arrhenius plot of *ln* k_2 versus *1/T* was 30.4 kJ mol^{-1} (Fig. S4).

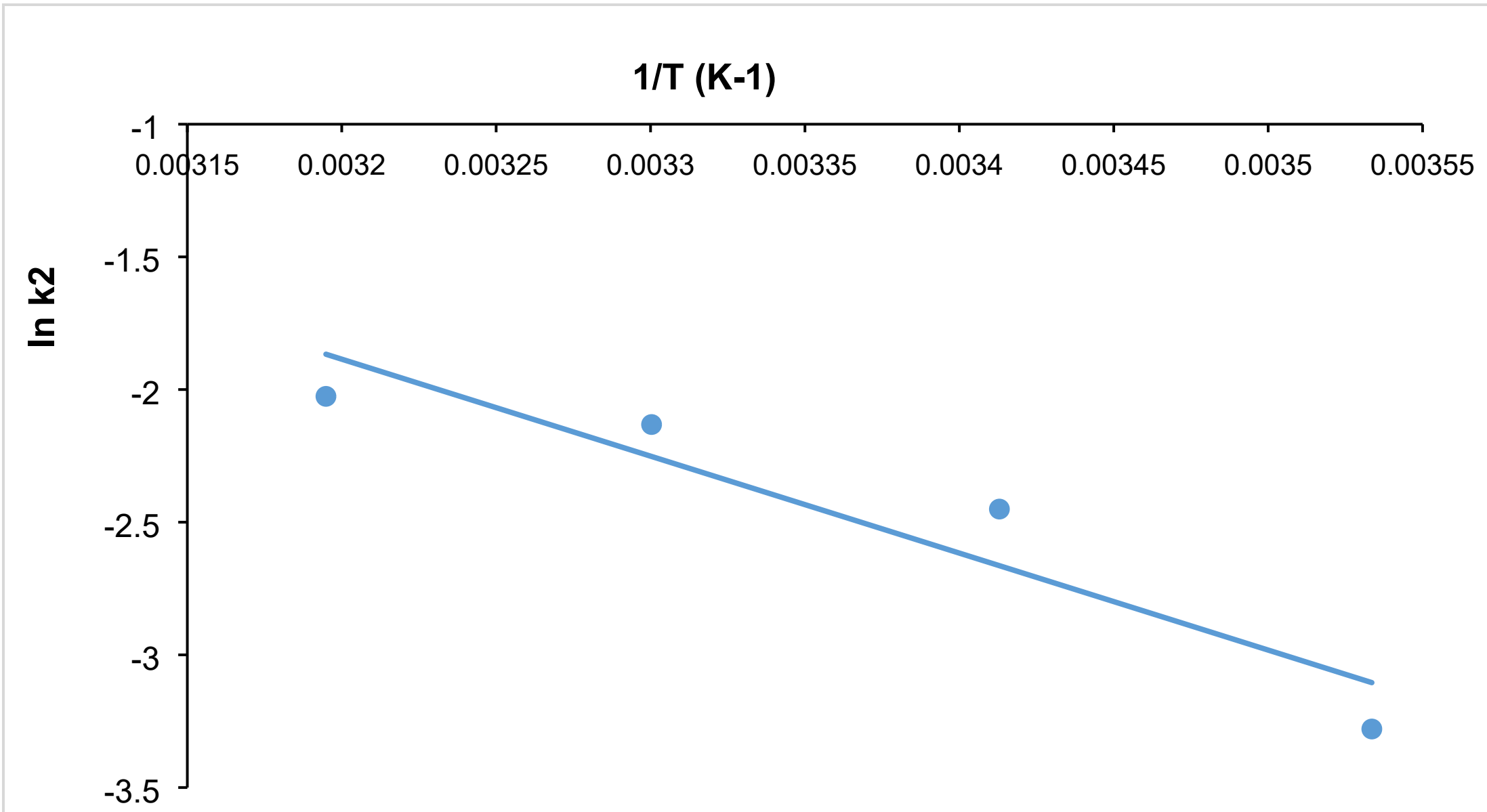

Fig. S4. Arrhenius plot of ln k_2 versus 1/T.

S5. Fixed-Bed Column Performance Analysis and Thomas Breakthrough Model

This section presents the numerical breakthrough dataset, column-performance calculations, and Thomas-model fitting used to support the fixed-bed analysis in the main manuscript. The representative run was conducted at a nominal influent PHY concentration (C_0) of 20 mg L^{-1}, a flow rate (Q) of 2.0 mL min^{-1}, and a packed adsorbent mass (m) of 3.0 g. The breakthrough curve was analyzed using the normalized effluent concentration (C_t/C_0) as a function of time.

S5.1 Experimental breakthrough data and calculation basis

The breakthrough time was defined at $C_t/C_0 = 0.10$, and linear interpolation between adjacent experimental points was used to estimate this value. The cumulative column capacity at any time t was calculated by trapezoidal integration using:

$$q(t) = (Q/m) \int_0^t (C_0 - C_t)\, dt = (Q\, C_0/m) \int_0^t (1 - C_t/C_0)\, dt$$

where Q was converted to L min^{-1} before calculation. A starting point of t = 0 min and C_t = 0 mg L^{-1} was used for integration, corresponding to an initially solute-free column effluent.

Table S5.1. Numerical breakthrough dataset used for the column calculations and Thomas-model fitting.

t (min)	C_t (mg L^{-1})	C_t/C_0	t (min)	C_t (mg L^{-1})	C_t/C_0
3	0.079	0.0040	48	5.680	0.2840
6	0.119	0.0059	51	7.909	0.3954
9	0.164	0.0082	54	11.661	0.5830
12	0.121	0.0060	57	13.674	0.6837
15	0.149	0.0075	60	15.386	0.7693
18	0.165	0.0082	63	16.467	0.8233
21	0.315	0.0157	66	16.727	0.8364
24	0.643	0.0322	69	17.115	0.8558
27	0.953	0.0476	71	17.245	0.8622
30	1.301	0.0650	74	17.372	0.8686
33	1.872	0.0936	77	17.335	0.8668
36	2.384	0.1192	80	17.500	0.8750
39	3.028	0.1514	83	17.631	0.8815
42	3.638	0.1819	86	17.707	0.8854
45	4.168	0.2084	89	17.838	0.8919

S5.2 Breakthrough capacity, saturation capacity, and bed utilization

Linear interpolation gave a breakthrough time (t_β) of 33.75 min at $C_t/C_0 = 0.10$, corresponding to a treated volume ($V_\beta = Q\, t_\beta$) of 67.50 mL. Integration to t_β gave an adsorption capacity at breakthrough (q_β) of 0.439 mg g^{-1}. At the final measured time of 89 min, C_t/C_0 was 0.8919 and the cumulative capacity was 0.723 mg g^{-1}. The experimental curve approached saturation but did not

directly reach the predefined exhaustion criterion of $C_t/C_0 = 0.95$ within the measured period. Therefore, the Thomas-model capacity ($q_{Th} = 0.758$ mg g⁻¹) was used as a model-derived saturation capacity. The bed utilization efficiency, calculated as $(q_\beta/q_{Th}) \times 100$, was 57.9%.

Table S5.2. Fixed-bed performance parameters for the representative PHY adsorption run.

Parameter	Symbol	Value / interpretation
Nominal influent concentration	C_0	20 mg L⁻¹
Flow rate	Q	2.0 mL min⁻¹ (0.002 L min⁻¹)
Packed adsorbent mass	m	3.0 g
Breakthrough time at $C_t/C_0 = 0.10$	t_β	33.75 min
Treated volume at breakthrough	V_β	67.50 mL
Adsorption capacity at breakthrough	q_β	0.439 mg g⁻¹
Final measured time	tend	89 min
Final normalized effluent concentration	C_t/C_0	0.8919
Cumulative capacity at 89 min	q89	0.723 mg g⁻¹
Experimental exhaustion at $C_t/C_0 = 0.95$	te	Not reached during the recorded run
Model-derived saturation capacity	q_{Th}	0.758 mg g⁻¹
Bed utilization efficiency	BUE	57.9% (= $q_\beta/q_{Th} \times 100$)

S5.3 Thomas breakthrough model

The Thomas model was selected as a representative fixed-bed model because it provides both a kinetic constant and a dynamic saturation capacity. The nonlinear and linear forms are:

$$C_t/C_0 = 1 / [1 + e^{[(kTh\ qTh\ m/Q) - kTh\ C_0\ t]}]$$

$$\ln[(C_0/C_t) - 1] = (k_{Th}\ q_{Th}\ m/Q) - k_{Th}\ C_0\ t$$

where k_{Th} is the Thomas rate constant (L mg⁻¹ min⁻¹) and q_{Th} is the Thomas dynamic adsorption capacity (mg g⁻¹). Regression of $\ln[(C_0/C_t) - 1]$ against time gave:

$$\ln[(C_0/C_t) - 1] = 5.8400 - 0.10270t \quad (R^2 = 0.9626)$$

From the slope ($-k_{Th}C_0$) and intercept ($k_{Th}q_{Th}m/Q$), the calculated parameters were $k_{Th} = 5.14 \times 10^{-3}$ L mg⁻¹ min⁻¹ and $q_{Th} = 0.758$ mg g⁻¹. The reconstructed Thomas curve gave $R^2 = 0.961$ and RMSE = 0.0736 for C_t/C_0. The model captured the overall sigmoidal breakthrough behavior, although the late tail remained broader than the ideal Thomas prediction, indicating non-ideal mass-transfer behavior in the granular bed.

Table S5.3. Thomas-model parameters for PHY adsorption in the Mnt/sand fixed bed.

Thomas parameter	Symbol	Calculated value
Linear regression equation	—	y = 5.8400 − 0.10270t
Thomas rate constant	k_{Th}	5.14×10^{-3} L mg⁻¹ min⁻¹
Thomas dynamic capacity	q_{Th}	0.758 mg g⁻¹
Linearized correlation coefficient	R^2	0.9626
Normalized-curve correlation coefficient	R^2curve	0.961
Normalized-curve RMSE	RMSE	0.0736

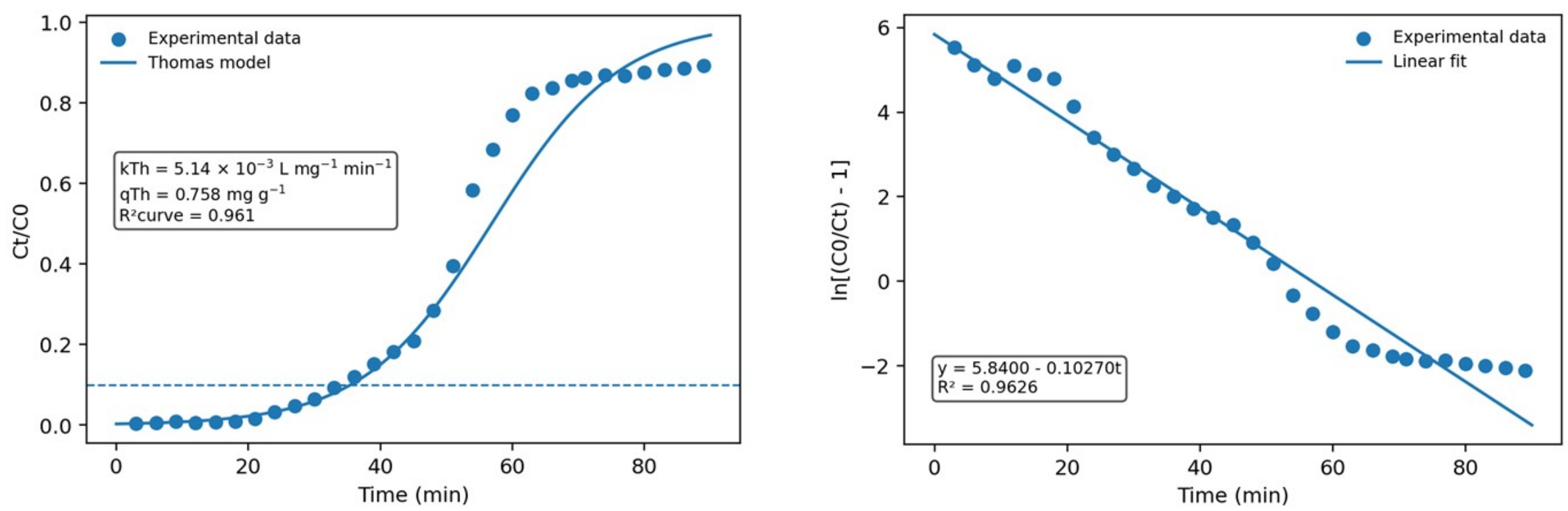

Figure S5. Thomas-model analysis of the representative fixed-bed run: (a) experimental normalized breakthrough data and Thomas-model prediction; (b) linearized Thomas plot.

The close agreement between the cumulative capacity measured at 89 min (0.723 mg g^{-1}) and the Thomas capacity (0.758 mg g^{-1}) indicates that most of the model-predicted capacity had been used by the end of the run. Because $C_t/C_0 = 0.95$ was not directly recorded, the model-derived capacity is distinguished from an experimentally observed exhaustion capacity. Longer column runs are recommended for direct confirmation of the 95% exhaustion point and for scale-up validation under additional flow rates, bed heights, and influent concentrations.

S6. DFT-Calculated Adsorption Energy for PHY Adsorption on Mnt

The adsorption energy of PHY on the Mnt surface was calculated from the DFT total energies using the following relationship:

$$E_{ads} = E_{Mnt/PHY} - (E_{Mnt} + E_{PHY})$$

where $E_{Mnt/PHY}$ is the total energy of the optimized PHY/Mnt adsorption complex, E_{Mnt} is the total energy of the clean Mnt surface, and E_{PHY} is the total energy of the isolated PHY molecule. A negative E_{ads} value indicates energetically favorable adsorption.

The total energies and calculated adsorption energy are summarized in Table S6. The calculated adsorption energy was −0.127310187 Hartree, corresponding to −334.25 kJ mol^{-1} or −3.46 eV. This negative value indicates that adsorption of PHY on the Mnt surface is energetically favorable under the adopted DFT model and supports the experimental evidence for strong PHY affinity toward the Mnt-containing composite.

Table S6. DFT-calculated total energies and adsorption energy for PHY adsorption on the Mnt surface.

Parameter	Symbol	Value	Unit
Energy of clean Mnt surface	E_{Mnt}	−552.022958	Hartree
Energy of isolated PHY molecule	E_{PHY}	−43.86170907	Hartree
Energy of PHY/Mnt adsorption complex	$E_{Mnt/PHY}$	−596.0119773	Hartree
Adsorption energy	E_{ads}	−0.127310187	Hartree
		−334.25	kJ mol^{-1}
		−3.46	eV
